# Changing readiness to mitigate *SARS-CoV-2* steered long-term epidemic and social trajectories

**DOI:** 10.1038/s41598-021-93248-y

**Published:** 2021-07-06

**Authors:** Kai Wirtz

**Affiliations:** Helmholtz-Zentrum Hereon, Geesthacht, Germany

**Keywords:** Infectious diseases, Viral infection

## Abstract

Societal responses crucially shape the course of a pandemic, but are difficult to predict. Mitigation measures such as social distancing are here assumed to minimize a utility function that consists of two conflicting sub-targets, the disease related mortality and the multifaceted consequences of mitigation. The relative weight of the two sub-targets defines the mitigation readiness ***H***, which entails the political, social, and psychological aspects of decision making. The dynamics of social and behavioral mitigation thus follows an adaptive rule, which in turn is mediated by a non-adaptive dynamics of ***H***. This framework for social dynamics is integrated into an epidemiological model and applied to the ongoing SARS-CoV-2 pandemic. Unperturbed simulations accurately reproduce diverse epidemic and mitigation trajectories from 2020 to 2021, reported from 11 European countries, Iran, and 8 US states. High regional variability in the severity and duration of the spring lockdown and in peak mortality rates of the first SARS-CoV-2 wave can be explained by differences in the reconstructed readiness ***H***. A ubiquitous temporal decrease of ***H*** has greatly intensified second and third waves and slowed down their decay. The unprecedented skill of the model suggests that the combination of an adaptive and a non-adaptive rule may constitute a more fundamental mode in social dynamics. Its implementation in an epidemic context can produce realistic long-term scenarios relevant for strategic planning, such as on the feasibility of a zero-infection target or on the evolutionary arms race between mutations of SARS-CoV-2 and social responses.

Societies struck by the severe acute respiratory syndrome coronavirus-2 *(SARS-CoV-2)* pandemics in early 2020, mostly in Western industrialized countries, managed to reduce infection rates through non-pharmaceutical mitigation such as social distancing^1,2^. After these societies started to lift lockdowns in May 2020^3,4^, some reached very low case numbers, while others faced continuously high mortality caused by the coronavirus disease 2019 (COVID-19). Later in autumn and winter 2020/2021, all these regions were hit by massive second and third waves, despite the experiences gained during the first lockdown^5^. By then, societies revealed considerable regional and temporal variation in their decision-making and application of mitigation rules^6,7^. One major problem when guiding the defense against *SARS-CoV-2* was the lack of reliable mid-term scenarios^8,9^, which stimulated the development of predictive tools. The greatest challenge in this development was the incorporation of human agency into conventional epidemiological models^7,10–12^, which could refer to few conceptual approaches^13,14^. Despite the high number of arising models based on, e.g., rule-based, fitted, extrapolated, or pre-defined scenario settings^9,15–20^ or immediate solution of an objective function^21–23^, none could so far reproduce the observed re-adjustments in social distancing across different countries.

Here, it is assumed that societal responses are more predictable than one commonly thought. Societal decision-making is in particular suggested to be rooted in rationality, which is expressed by a composite utility function that in turn becomes subject to non-adaptive changes. This means that variations of the response during a pandemic should reflect a region-specific balancing of mitigation costs and benefits with the weight of mitigation costs gaining priority over time, which increases societal avoidance and reduces mitigation readiness. The concept is integrated into a susceptible-infected-recovered (SIR) model for developing a generic because region-independent tool for mid-term epidemic predictions, which will here be used to run a number of numerical hindcast experiments. These experiments should test the plausibility of the concept insofar it is able to reconstruct regional variability in actual viral spread dynamics and in social distancing. The analysis of the experiments should then elucidate possible drivers and constraints of the social dynamics behind regional mitigation trajectories.

The model resolves seven age groups, and is the first to feature (age-specific) contact rates as prognostic and adaptive variables. Adaptive changes in social mixing underlying the *SARS-CoV-2* transmission are assumed to be driven by three pressures describing the benefits and costs of social distancing: (1) individual avoidance of one’s own infection and mortality, (2) social coherence in reducing the overall infection levels, and (3) costs of social distancing such as financial and mental health losses (see derivation in “Material and methods” section). Changes in contact rates are then determined by the balance of pressures (1)–(2) representing the benefit of less mixing and of the pressure (3) induced by the costs of less mixing. The pressures are associated with COVID-19 mortality *M* in cases (1)–(2) and with the multifaceted socio-economic consequences *C* in case (3). They induced transmission shifts that minimize integral social and mortality costs ($M+C$): during a pandemic, optimal contact rates are greatly reduced compared to business-as-usual (BAU) social mixing (Fig. 1a). When integrating *M* and *C* in the same metric, the model introduces a social trait that quantifies the relevance of avoiding deaths versus keeping BAU contact rates, thus representing a “human value”. This trait describes a full suite of aspects and dimensions in societal decision-making: the priority of governments to safeguard the economy, their facilitation of partisan polarization^24^, capacity of elites and people to extrapolate in time (see Sec. S2), presence of misinformation and scepticism versus effective science communication^12,25^, group (in-)coherence and (non-)conformity to norms^25^, individualistic versus community oriented norms^25^, psychological resilience versus fatigue^25,26^, or other individual attitudes such as patience, altruism, and trust in institutions^12,25,27^. These aspects can be mutually dependent, such as norm adherence of individuals being linked to socio-economic inequalities^26^. In summary, the aspects determine the *mitigation readiness* of a society during a pandemic as quantified by the “human value” *H*. Societies characterized by a low mitigation readiness *H* tolerate a higher death toll before restricting mixing and mobility compared to those with a higher *H*. At low *H*, elevated social costs *C* curtail social distancing to small deviations from the BAU baseline (Fig. 1b). The mitigation readiness *H* is the only adjustable parameter of the model to address regional differences in response to *SARS-CoV-2* throughout the entire simulation period. It is treated differently within two model variants: either *H* is kept constant at a base value $H_0$, or it steadily declines after the first lockdown from $H_0$ at the disintegration rate $r_H$.

**Figure 1:**
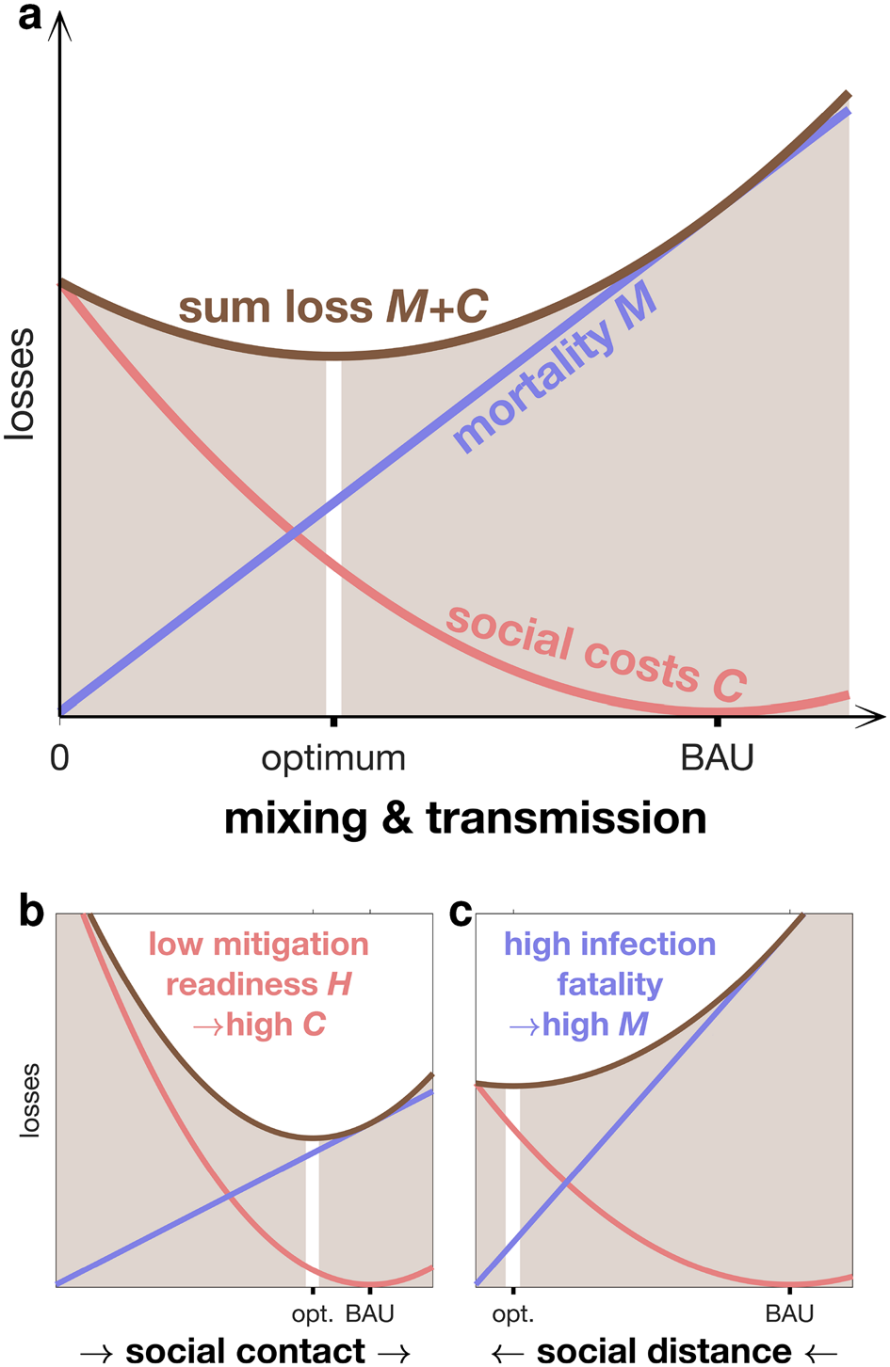
COVID-19 associated losses depending on contact rate. (**a**) Social costs *C* (red line) including, e.g., economic downturn, cultural loss, political instability, and psychological pressure, in crease with social distancing and less mixing. The specific increase is high at low readiness *H*, thus a low value of human lives versus social contacts. *C* is assumed to have a minimum at “business-as-usual” (BAU) contact rates and to increase non-linearly with growing distance from those BAU contact rates; mortality *M* (violet line) linearly increases with contact rate. The minimum of the sum loss $M+C$ (brown line) is in the model approached by an adaptive adjustment in social mixing. (**b**) Younger societies will often feature a lower *H* due to lacking buffer mechanisms and lower fraction of people at risk compared to older societies. The resulting high social costs of social distancing keep the contact rate close to the BAU value. (**c**) In contrast, aged societies will have on average a higher infection fatality ratio and, concomitantly, mortality rate, which motivates stricter lockdowns.

In addition to the adaptive contact regulation, driven by utility, and to the non-adaptive disintegration of readiness, the model resolves adaptive changes in individual behavior. These behavioral changes also depend on environmental factors. For example, moving everyday life outdoors during summertime or the wearing of face masks can effectively reduce exposure to viral infection (see “Material and methods” section).

This study considers the COVID-19 associated mortality rate not only as part of the utility function but also as a major variable used for validation. Mortality data make a more reliable indicator for the infection state than the number of confirmed cases^9,28,29^. Selected by their high mortality rates in spring 2020, 20 regions were examined in this study, comprising 11 European countries, Iran, and 8 US states (see Tab. S1 and “Material and methods” section).

## Model skill

**Figure 2:**
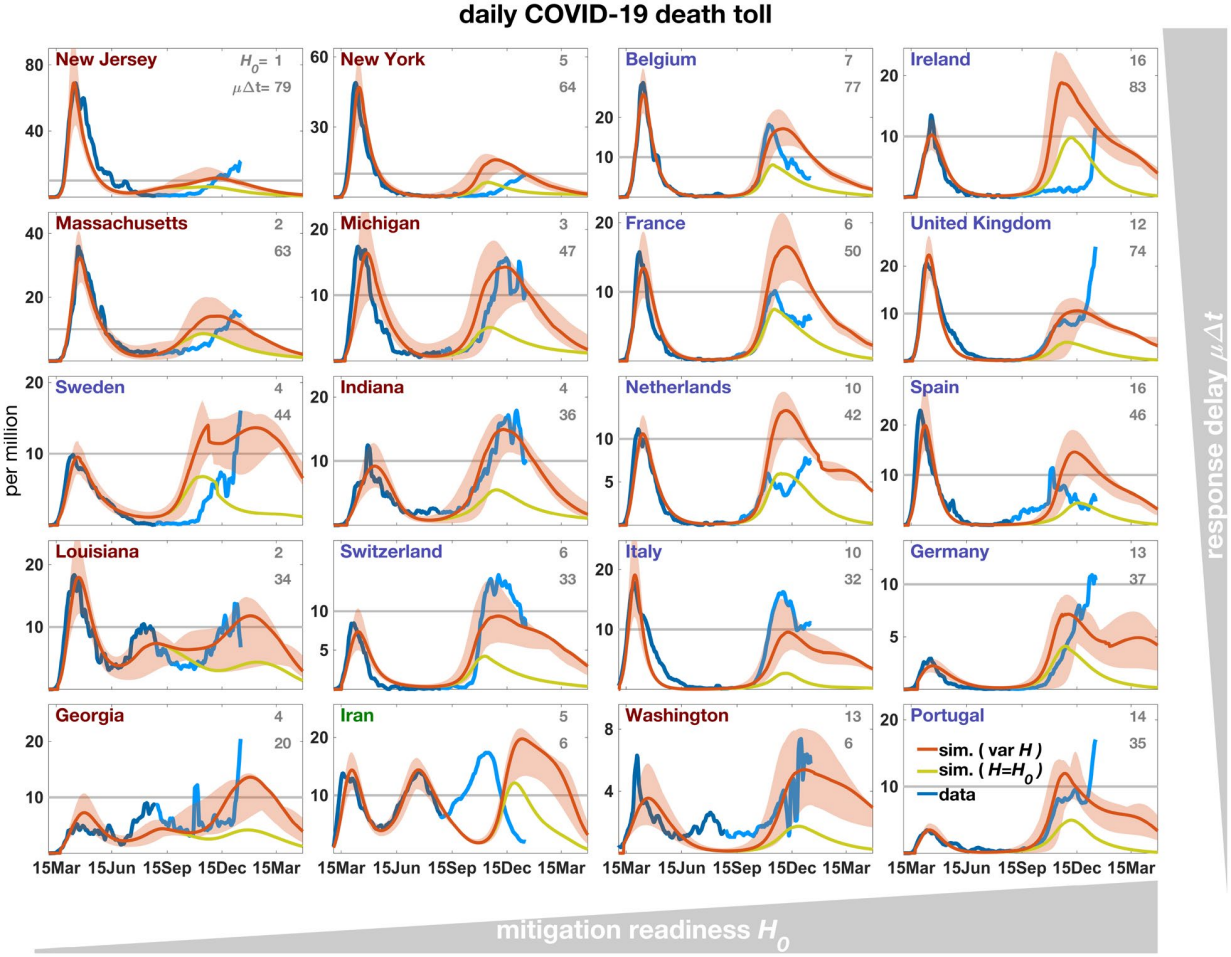
Daily mortality rate simulated either in a variant with constant *H*=$H_0$ (olive line, $r_H$= 0 in Eq. (4)) or one with decreasing *H* ($r_H>$0) after the first lockdown (red line). Uncertainty in model trajectories (shaded areas) arises from simulations with close-to-optimal $H_0$ values as well as a range in external input ($\gamma$). From the reported and corrected mortality data (see “Material and methods” section, blue line) only the first 180 entries were used for calibration of $H_0$ (dark blue line), while the second half of the time-series is shown for comparison (light blue line). Note the different scaling of the y-axis as also visualized by the grey line at *M*=10$^{-5}$d$^{-1}$, which roughly corresponds to the mortality rate at starting capacity limitation of ICU hospitalization. European countries are labelled in blue, US states in red. The ordering of regions from left to right reflects increasing base $H_0$ (defined in Eq. (5); grey numbers to the right top, relative to 10$^{4}$), and from top to bottom the decreasing product of the initial spread rate $\beta _0'$ Sec. S5 and the awareness $\Delta t$ (Sec. S2).

Across the 20 regions, simulated COVID-19 associated death counts were consistent with the data (Fig. 2). Simulated and reported mortality accurately match for the period from February to September 2020, and also the subsequent wave was reproduced by the model with only moderate deviations and time lags, including the occurrence of third waves for Louisiana, Georgia, and Iran. Fitting of the second and third waves can be further improved by calibrating three instead of one parameter (Fig. S2). The overall agreement is remarkable because mortality trajectories differed greatly among regions^9,28^ and model runs represent true hindcasts: apart from a superimposed synchronous initiation of the first lockdown (see “Material and methods” section) simulations were not corrected or tuned. This indicates a high predictive capability even in the mid to long term.

## Lockdown severity and mitigation readiness

**Figure 3:**
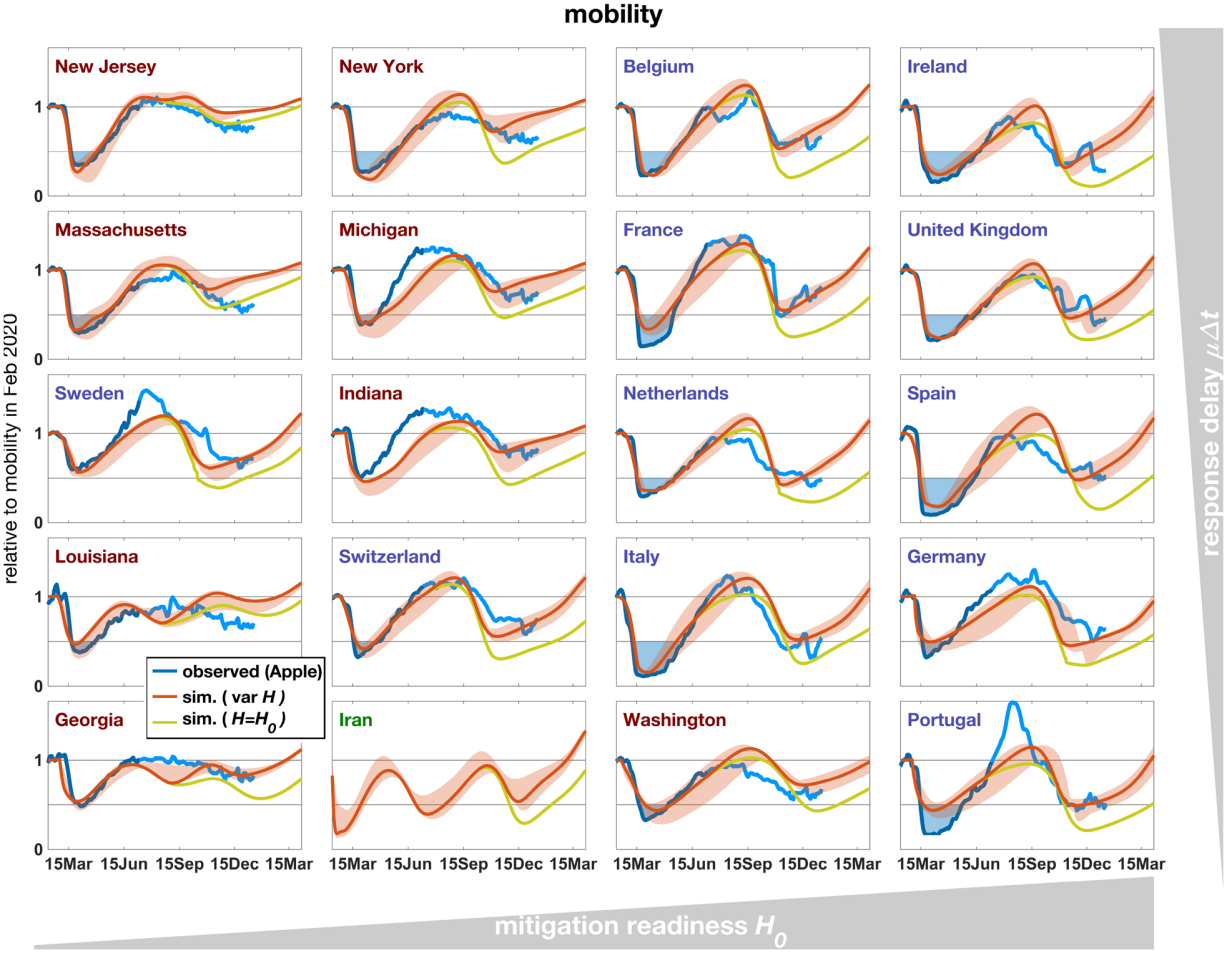
Mobility in 2020–2021 measured based on routing requests from mobile Apple devices (blue line), compared with the summed contact rate (Eq. (S8) in Sec. S6) in the reference simulation with constant *H*=$H_0$ (olive line) and the simulation with decreasing *H* (red line, see Fig. 2). Severity of the spring lockdown is displayed as blue area below a mobility of 50% of the base level in February 2020.

**Figure 4:**
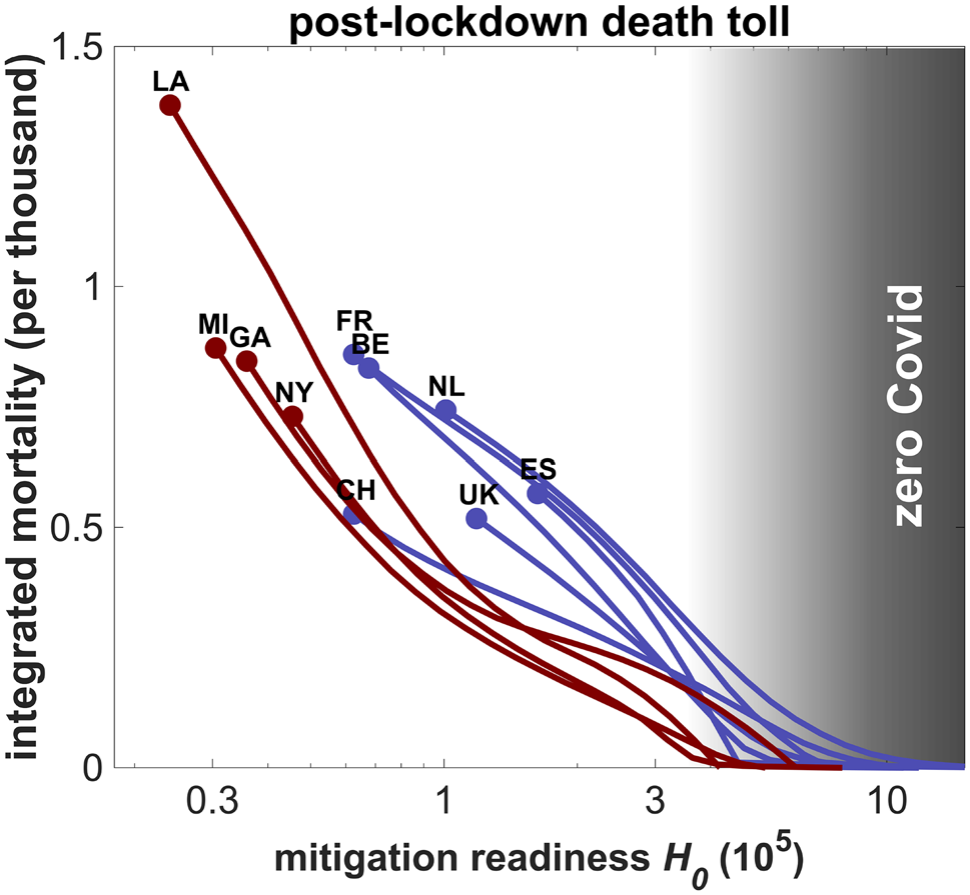
The death toll after the first lockdown for increased base mitigation readiness (with constant *H*=$H_0$). The grey area marks the range of $H_0$ corresponding to mitigation efforts that are required to eradicate the virus (“zero Covid”). Reference $H_0$s are highlighted as circles. Travel input and disintegration were switched off ($\gamma '$=0, $r_H$=0). For better visibility, only half of the regions are shown; their abbreviations are given in Tab. S1.

The high skill of the model largely relied on the account of human agency as visible from the stark differences between the reference run and a variant neglecting mitigation and behavior (Fig. S1). The ratio between the reported intensity of social distancing and mortality during the first wave constrains regional values of the base mitigation readiness $H_0$ (see Eq. (5) in “Material and methods” section). These reconstructed differences in $H_0$ already account for regional variations in the age structure. For example, the typically high median age of European populations (Tab. S1) infers a high infection fatality ratio (IFR) and therefore also severe social distancing at otherwise equal specific mitigation costs as given by $H_0$ (Fig. 1c). Notwithstanding, high $H_0$ were calibrated for many European countries that had faced a strong and enduring spring lockdown (Fig. 3) independent of their peak mortality rate (Fig. 2). To the contrary, inverse modeling attributed a relatively low $H_0$ for most US states with their often milder lockdowns despite elevated mortality (Fig. 2, S3, Tab. S1). Values for US states, apart for Washington, lay in a narrow range (1.3–4.2 10$^{4}$), which may point to a small variability of this aggregate social trait within countries (see also Fig. 4). Regional differences especially in the trajectories during spring–summer were not only determined by calibrated $H_0$, but also by the variable age and household structure. For example, minimal contacts already found earlier to shape the first wave^21^ are here formulated in terms of the household structure (Sec. S6). In regions with small $H_0$ and lacking intense first lockdowns, mortality either decayed much slower compared to the average of all regions such as in Sweden, or a second wave built up already in summer 2020 such as in Louisiana (Fig. 2). The simulations well captured not only regional differences in lockdown severity, comprising a lockdown mobility above 50% of pre-pandemic levels (e.g., in Sweden or Georgia) or below 20% (e.g., UK or Italy), but also the different rates of recovery in mobility such as a fast return to BAU mobility in New Jersey versus a slower one in Washington (Fig. 3). The single calibration parameter $H_0$ hence appeared to infer a realistic mutual interdependency of mobility and mortality patterns across regions so that mobility trajectories were overall in high quantitative agreement with the data.

## Decreasing readiness promoted the second wave

For the second or third waves, the agreement between reported and simulated trajectories deteriorates if mitigation readiness stays constant in the model. Peak mortality rates in autumn 2020 found by hindcasting were on average a factor of three lower than the reported ones. Peak death tolls were instead quantitatively reproduced by the model variant including a catch-up mechanism generated by a steady post-lockdown decline in *H* (disintegration rate $r_H>0$, Fig. S3); reproduced peaks in part had a temporal shift of up to 10 weeks, such as for Ireland where data of late January (not shown) agree with the forecasted peak height (Fig. 2). The second wave was better fitted by the first model variant in France and the Netherlands, however at the cost of overestimating mobility in winter 2020/2021 (Fig. 3). After extending the regional calibration to more parameters, the COVID-19 mortality rates of these countries were best reconstructed using non-zero disintegration rates (Fig. S2). The model variant with $r_H>0$ in general reproduces the strong social mixing, happening during late 2020, in the data more accurately than the variant without disintegration (Fig. 3). Better performance of the variant with $r_H>0$ is also found in the third waves in Louisiana and Georgia (Fig. 2).

In Louisiana and Georgia—and a few others—peak mortalities were underestimated by an extensive US-wide modeling study^20^. There, simulations were well constrained also in terms of mitigation measures until autumn 2020 and run freely thereafter. This, together with the much better fit of the model presented here in its extended calibration (allowing for later disintegration start, Fig. 2, S2), point to a rather delayed decline of mitigation readiness in some regions (e.g., Louisiana and Georgia). The simple scheme proposed here (Fig. S3) thus requires refinements such as a dependency of the disintegration on social or psychological factors.

## Alternative pathways for industrialized countries

The moderate autumn/winter death toll in the model variant with constant *H*=$H_0$ raises the question as to whether different mitigation strategies in the study regions could have led to a “zero Covid” situation, as achieved by few Asian countries, and also proposed as best-case scenario elsewhere^30^. More rigid strategies were here emulated by upwards shifts *H* after the first lockdown (and then keeping *H* constant) in consecutive numerical experiments. After raising readiness *H* by about one order of magnitude from the regional base value, viral infection was eradicated across regions (Figs. S4, 4). One may argue that Western societies may not have tolerated deeper and longer cuts into individual rights of privacy and movement or into economic operations at nearly invisible infection density in summer-autumn 2020. However, magnitudes of the upwards shifts in *H* required for a practical extinction of the pathogen correspond well to the magnitudes of (dynamic) downward shifts reconstructed for the same period (Fig. S3). The corresponding high “human value” ($H>10^6$, Fig. 4), hence, does not entirely appear out-of-reach and may reflect not only higher appreciation of each individual life but also greater awareness of the exponentially growing number of cases following each infection.

Along these lines, an otherwise non-preventative strategy or a full travel ban cannot improve the situation by very much. To the contrary, without imported cases, $\gamma$=0 in Eq. (1), simulated peak mortality rates of the second wave even increased in regions with low case numbers during summer (Fig. S5). When viral infection strikes from very low non-zero levels achieved at $\gamma$=0, spreading rates can develop faster compared to the reference scenario ($\gamma >0$). Yet, faster spreading rates are harder to defend against, which evokes higher peak mortality rates.

## Role of young people

Social distancing similarly affected all age groups. Simulated age distributions of cases were rather flat (Fig. S6), in qualitative agreement with first seroprevalence studies^31,32^. The imposed decline of BAU contact rates from the younger and the elderly is counterbalanced by the model setting of lower attack rates of the younger. As a result, young and medium aged cohorts can maintain finite contact rates during the lockdown, especially in low *H* regions such as the US (Fig. S7 and Fig. S6). Contagion within younger adults during summer 2020 fueled the hindcasted epidemic rebound in all study regions (Fig. S8). The low IFR of young adults also explains why according to reported data the ubiquitously higher case numbers of the second wave (Fig. S8) coincided with lower mortality in most regions^33^.

The shift toward younger ages during summer is confirmed by US and German monitoring data^34,35^; although, the cohort from age 15 to 30 (year) was the most prominent, whereas simulated infection levels were highest among adults older than 30 (Fig. S6). This discrepancy may indicate a lower conformity with mitigation measures within young cohorts than assumed by the optimal transmission regulation of the model, which is corroborated by studies revealing stronger non-conformist attitudes among adolescents and young adults during the pandemic^36–39^.

## Behavior and seasonality matter

During the course of the spring lockdowns, decreasing behavioral exposure $e_b$ significantly helped to combat the first wave in the simulations (see also Fig. S10), a finding that underlines the relevance of using face masks^40,41^. Readiness to improve behavioral protection appeared to increase under high peak mortality and/or high *H* value since both conditions cause intense (model) lockdowns that are here linked to behavioral shifts.

Even in regions displaying relatively inert behavioral adaptation, effective exposure *e* (= $e_b\cdot e_E$) markedly decreased in late spring 2020, which in the model follows from the transition to spread-reducing environmental conditions ($e_E$). The decreases in $e_E$ condense multiple bio-physical and behavioral processes driven by higher temperature and intensity of solar radiation, such as the effects on viral viability, or on shifting activities from indoor to outdoor. Conversely, as also anticipated by virologists^42,43^, returning autumn/winter conditions contributed to the arrival of the second wave (Fig. S10), which is also visible from the synchronized dynamics of *e* and *I* (Fig. S9 and S8). Seasonality effects are, against expectation, most evident for regions at relatively low latitude such as Louisiana and Iran, where the increase in $e_E$ already started during summer (Fig. S9) due to supra-optimal temperatures, and was furthermore accompanied by high values of behavioral exposure $e_b$ (see also Fig. S10). $e_b$ relaxes to higher values in all regions (Fig. S9) during summer, which seems to contradict the constant or later even increasing overall willingness to wear face masks as suggested by polls^44^ (redrawn in Fig. S9). This relaxation is in part a model artifact linked to the simplistic coupling of changes in behavior with social distancing. It may however also emulate a real mechanism of declining protection due to higher behavioral exposure of young people^36–39^. Given their increasing dominance of the case distribution, this has likely induced a net shift in averaged $e_b$ towards less protective behavior despite the overall trend towards higher protection.

In total, higher behavioral as well as environmental exposure together with softer social distancing in winter 2020/2021 considerably slowed down the decay of the second wave in comparison to the first wave (Fig. 2).

## Increasing the forecast horizon

In all simulations, infection waves were dampened or halted by transmission reduction and not by depletion of those susceptible, as forecasted by many state-of-the-art models. SIR models cannot seamlessly produce flat infection curves if they do not account for human agency (see also Fig. S1). This in part explains why—similar to similar statistical models–SIR models typically have a forecast horizon of only few weeks^8,45,46^.

First attempts to extend epidemiological dynamics by macroeconomic factors^27,47–49^ use a utility function similar to the approach presented here; they also distinguish between different types of agents such as “private individual” (cf. here the selfish pressure) or “social planner” (community pressure). However, economic models rely on equilibrium assumptions and on strictly quantifiable (monetary) units and, thus neglect potentially important non-economic aspects of societal decision-making such as learning under uncertainty, psychological fatigue, or political partisanism^12,24,25^. The negligence of sensible and dynamic control processes may be responsible for why the regular outcome of economic approaches remained within the herd immunization scenario of SIR models. Much related to these economic models, optimal control^21–23,50^ or game theoretical approaches^51^ use an objective function similar to the one used here but assume instantaneous optimization. By contrast, the adaptive dynamics approach taken here resolves finite flexibility and (delayed) learning of societies. At higher responsiveness, thus approaching an idealistic “optimal control” scenario, secondary and third waves would have been much more suppressed (see Louisiana at higher $\delta$ in Fig. S10). Another important difference to previous models accounting for behaviour is the integration of age-structure, which however necessitated to combine altruistic and self-interested aspects in the objective function. The results shown here overall suggest the high relevance of models to resolve societal responses dynamically.

Other than social *dynamics*, recent approaches emphasize social *actions*; they are based on semi-heuristic rules of social distancing such as piecewise re-fitting of transmission^19,52^, imposing pre-defined or rule-basedshifts^17,18,20^, relaxing transmission^28^, and relaxation cycles^9,15^. These approaches may be a supportive tools for short-term decision problems, but often lack a testable description of (changing) mitigation as well as a validation of a monthly or longer time scale. More validation effort is also required for the model presented here; This could include application of the model to a broader range of regions, in particular non-Western countries. As for any model, there are caveats, which are briefly summarized in Sec. S9. Yet, the ability to hindcast rich variability in mitigation and mortality trajectories across regions can be exploited for supporting planning against forthcoming epidemic waves. While some regional characteristics such as age structure or seasonality cannot be shifted, behavior can change, however at multiple costs with mid- to long-term repercussions. Policy makers and the public could be informed on consequences of staying at or relaxing from a certain readiness level. This study should in particular increase their awareness about the relevance of ongoing social dynamics. Given that the decline in readiness has been suggested here as the primordial driver for the evolution of mitigation and viral spread in western countries from autumn 2020 to spring 2021, mitigation efforts should be planned at a longer time horizon, which is here defined by the availability of pharmaceutical measures such as vaccines. In the face of a pandemics, societies can either sprint to a “zero Covid” target—or run at a pace they can hold over an enduring battle.

## Blueprint for adaptation problems

This study highlights adaptive social responses, individual behavior and their possible deterioration as critical controls of the *SARS-CoV-2* pandemic. The unprecedented flexibility of the model to fit epidemic and aggregated contact curves across many regions may indicate that the model captured the governing principles of viral and social dynamics during a pandemic to a reasonable degree and that adaptive capacity makes an important component of human agency.

However, adaptive capacity is also an attribute of viruses. Mutations in *SARS-CoV-2* started to impact spread trajectories^53–56^. These mutational drifts in parameters of *SARS-CoV-2* virulence and incubation behavior can be resolved analogue to the adaptive dynamics implemented in the social model (Eq. (7) in “Material and methods” section). This extended framework would facilitate modeling studies on the evolutionary arms races between human societies and *SARS-CoV-2* or other viruses. The framework can further be used as a blueprint for related problems, such as Climate Change assessments, which share, e.g., the balancing of environmental pressures with costly adaptation and mitigation efforts, or the need for extrapolating aspects of the utility function into the future. During the pandemic and Climate Change, human agency is not an external boundary setting but an integral part of system dynamics.

## Material and methods

### The societal-epidemiological model

The epidemiological section of the model resembles a SIR model as it distinguishes between susceptible and recovered people, and those infected by *SARS-CoV-2*. For seven age classes $i=1\ldots 7$, it resolves the fraction of infected individuals $I_i$ of age group *i* relative to the total population size. $I_i$ increases when susceptible people in that age class ($S_i$) contract the virus and decreases at specific recovery rate *r* (Tab. S2):

1 $$\begin{aligned} \displaystyle \frac{\text {d}I_i}{\text {d}t} = \beta _{i}\, S_i - r\, I_i + \gamma _i \qquad \text {with}\quad \beta _{i}=e\,\sum _j \beta _{ji}{I}_j \end{aligned}$$

A global external input rate $\gamma$ into a region (e.g., from travelers) is parametrized in Sec. S7. At simulation start, the fraction of susceptible individuals $S_i$ equals the population fraction $\varphi _i$ of the age cohort and thereafter declines due to infection and subsequent immunization or fatality, $S_i = \varphi _i\,{\mathrm {e}}^{-\int {\beta _i}dt}$. Group transmission rates $\beta _i$ comprise (1) variations in the effective exposure $e=e_b\cdot e_E$ by behavioral changes $e_b$ and environmental factors $e_E$ (see below and Sec. S8) and (2) changes in contacts between age cohort *i* and all age groups ($\sum _j \beta _{ji}{I}_j$). The specific transmission rate $\beta _{ji}$ describes the probability *per individual* of potentially *contagious* encounter, and has to be distinguished from the contact rate $m_{ij}$, which is the probability *per age group* of *physical* encounter,

2 $$\begin{aligned} \varphi _i\beta _{ij} = \alpha _i \alpha _j m_{ij} \end{aligned}$$

with specific attack rates $\alpha _i$ (Sec. S1). Infection described by Eq. (1) leads to a (lagged) mortality rate *M* caused by COVID-19 given by

3 $$\begin{aligned} M = \sum _i \omega _i\,\beta _{i}\,S_i \end{aligned}$$

with age-specific IFR $\omega _i$ (Sec. S1).

Reductions in mixing and transmission by social distancing or other related restrictive measures induce a multi-facetted “social cost” (*C*)^57^. This quantity aggregates over various damages of social distancing on economic and psychological well-being, political stability, or cultural diversity^1,16,57–59^. Social cost *C* of mitigation is assumed to rise with increasing social distance ($\text {SD}$), which is the sum over all differences of contact rates $m_{ij}$ to the associated values $m_{ij,0}$ before the epidemic, weighed by $m_{ij,0}$ and sizes of interacting age classes.

4 $$\begin{aligned} C= H^{-1}\cdot \text {SD} \qquad \text {with}\quad \text {SD}=\sum _i\sum _{j\le i}\varphi _i\,\varphi _j\,m_{ij,0}\cdot \Bigg (1-\displaystyle \frac{m_{ij}(\beta _{ij})}{m_{ij,0}}\Bigg )^2 \end{aligned}$$

The quadratic dependency on contact rate ratios (being linearly related to *M*) resembles the relationship between GDP loss and mortality at varying social distancing parameters formulated in few economic models^48^or the one between reduction in transmission rate and the objective function in an optimal control model^23,50^. The quadratic relationship encompasses tolerance against small deviations and the strong effects of downturning contacts to their minimum. Similar functions were recently shown to induce only moderate differences in the model results, unless they would be linear in $m_{ij}(\beta _{ij})$^23^. The proportionality coefficient $H^{-1}$ determines how mitigation costs at a given level of $\text {SD}$ are perceived and prioritised by a society in units of the mortality rate so that its inverse, *H*, is here denoted as mitigation readiness since a low specific weight of costs is connected to high willingness to mitigate. The specific burden or weight $H^{-1}$ may grow over time because of delayed and accumulating impacts of mitigation on societal, economic, and psychological well-being^58–61^. Inspired by classic models for smooth transitions between two socio-technological states^62,63^, the time evolution of $H^{-1}$ is assumed to follow a logistic growth equation

5 $$\begin{aligned} \displaystyle \frac{\text {d}\,iH}{\text {d}t} = r_H \cdot iH \cdot \Bigg (1-\displaystyle \frac{iH}{c_H}\Bigg ) \qquad \text {with}\quad iH=H^{-1}-H^{-1}_0 \end{aligned}$$

with “disintegration” rate $r_H$, lower boundary $H^{-1}_0$ ensuring finite social costs, and upper boundary (“capacity”) $H^{-1}_0+c_H$. In the model, the disintegrative increase of $H^{-1}$ is activated at day $t_{\mathrm {reset}}$ when the net infection at low case numbers returns from a negative to a positive rate after the first lockdown. The analytical solution of Eq. (5) is a sigmoid function, ${H} = {H}_0 \cdot (1+c_H/2)\cdot (1+\,{\mathrm {e}}^{-x})(1+c_H+\,{\mathrm {e}}^{-x})^{-1}$ with $x= r_H\,(t-t_{\mathrm {reset}})$.

A zero or non-zero disintegration rate $r_H$ distinguishes the two model variants used in this study. Mitigation readiness *H* links the socio-economic part of the model with the epidemiological one because it converts the loss function *C* to the same units as the loss function *M*. This enables the definition of the total loss *L* function,

6 $$\begin{aligned} L = C + M \end{aligned}$$

Avoidance of pathogenic transmission (by lowering $\beta _{ji}$) and, as a consequence, reduced COVID-19-associated death toll has to be traded off with associated social costs. Societal transmission regulations are suggested to be rational, in terms of minimizing the combined loss *L*. The existence of the utility function $L(\beta _{ij})$ describes the social regulations as the adaptive dynamics of specific transmission rates $\beta _{ij}$. Following the adaptive trait dynamics approach^64^, also applied to societal dynamics^65^, transmission regulation is formulated as an evolution equation for $\beta _{ij}$, as a result emerging from decisions of individuals up to the “central planner”. Direction and finite speed of this multi-level regulation entails a “responsiveness” $\delta$ times the marginal dependence of *L* on changes in $\beta _{ij}$.

7 $$\begin{aligned} \displaystyle \frac{\text {d}\beta _{ij}}{\text {d}t} = - \delta \cdot \displaystyle \frac{\text {d}L}{\text {d}\beta _{ij}} =- \delta \cdot \Bigg [ \displaystyle \frac{\partial C}{\partial \beta _{ij}}+\displaystyle \frac{\partial M}{\partial \beta _{ij}} + \displaystyle \frac{\text {d}M}{\text {d}I}\displaystyle \frac{\text {d}I}{\text {d}\beta _{ij}} \Bigg ] \end{aligned}$$

In a physical analogue, responsiveness $\delta$ describes the conductivity of how fast emerging threats induce new societal rules. The bracketed derivative terms then express a pressure acting on social traits, which is divided into three parts (see also Sec. S2): the first term in Eq. (7) can be directly calculated from Eq. (4) to be proportional to $\beta _{ij,0}-\beta _{ij}$ and hence seeks to relax societal life to the pre-pandemic state. The second term in Eq. (7) quantifies the demand of life protection and simply follows from the mortality dependence on infection rates in Eqs. (1)–(3). This term is proportional to the IFR $\omega _j$ of the target age group, which strongly decreases in younger cohorts (Sec. S1). As a consequence, only interactions with and among senior groups would experience high reduction pressure; however, these contacts among or with the elderly cannot be shut down entirely (see Sec. S6), so that virulence among young people can persistently contaminate the elderly. This side effect of isolated regulation in individual age-groups necessitates the extension of an adaptive dynamic framework by the third, “community-oriented” derivative term in Eq. (7), which is based on averaged target variables (*I* instead of $I_i$). This term represents the responsibility of governments and the population as a whole, and requires sociality of young, non-risk groups (Sec. S2).

In addition to the adaptive shifts in contact rates, the model includes variations in the behavioral reduction of exposure $e_b$. For example, wearing face masks or keeping sufficient interpersonal spatial distance up to self-isolation further lowers the infection risk at a given frequency of physical contact. The difficulty in formulating a reasonable cost function for behavior changes leads to a heuristic dynamics linked to social distancing ($\text {SD}$, defined in Eq. (4)): people are assumed to be more prone to adopt new behavioral rules at higher reductions in mobility and livelihood. This is expressed by a relaxation where $e_b$ seeks to approach a target value $e^*$ that decreases from its pre-pandemic value $e^*$=1 with increasing $\text {SD}$

8 $$\begin{aligned} \displaystyle \frac{\text {d}e_b}{\text {d}t} = r_b \cdot \left( e^* - e_b\right) \qquad \text {with}\quad e^* = 1-\epsilon \cdot \sqrt{\text {SD}} \end{aligned}$$

with specific adoption rate $r_b$ and specific behavioral sensitivity $\epsilon$. The square root dependency reverts the squaring in Eq. (4) in order to create sensitivity to small variations in $\text {SD}$.

## Data integration and region selection

Fatality data were downloaded on Jan, 16, 2021 from the Johns Hopkins CSSE COVID-19 Dataset^33^ and smoothed by 7-day averaging. A regional correction factor was then applied to the data. The factor averages the temporal means of the CSSE data and the estimated excess deaths for US states^66^ and for European countries^67^. Regions were selected if they had >700 death cases by April 25, 2020, and a relative mortality above the threshold $M_{\mathrm {crit}}$ = 7$\cdot$10$^{-7}$d$^{-1}$ by Mar, 25, 2020. China was excluded due to data irregularities and to its pioneering role in handling the epidemic. For Ireland, old cases from retirement homes reported on April 24 were re-distributed to the preceding time series. Iranian mortality data were multiplied by a higher and initially dynamic correction factor to comply with media reports^68^. Tab. S1 provides a full list of countries and states, correction factors, and demographic or regional characteristics.

For all study regions except for Iran, mobility has been reported from routing requests of Apple mobiles^69^, which is taken as a measure for the intensity of social distancing^70,71^. For 7 of the selected European countries and USA at the country level, survey data on the willingness to wear face masks in the public^44^ were used as a qualitative proxy to compare with simulated changes in behavioral exposure.

## Numerical experiments

This study is based on two systematic model calibrations (A, C) and three numerical experiments (B, D–E):

(A) For each region, the model was run over 400 days from 21 days before the date when the reported daily mortality reached $M_{\mathrm {crit}}$. Initial cases $I_i(0)$ were set proportional to (1) the regional age distribution $\varphi _i$ and (2) the critical onset mortality $M_{\mathrm {crit}}$. Initial transmissions $\beta _{ij}(0)=\beta _{ij,0}$ were derived from reported age—contact data and corrected using the slope of the mortality curve at the start of the simulation (Sec. S5). The social trait *H* and the awareness factor $\Delta t$ (Sec. S2) were systematically varied in 800 simulations for each region. Epidemiological parameters were estimated from literature sources (see Sec. S1). The calibration of $\Delta t$ assured a rather synchronous lockdown timing of Western industrialized countries in mid-March^72^. The reported lockdown onset was anticipated by one week for Italy and Iran, and delayed by 5 days for all US states. Best fitting $H_0$s were retrieved according to minimal root-mean-squared (RMS) deviation to mortality and mobility data while only the first 180 days of data were used. $H_0$ values revealing a RMS error below 120% of the minimum were used to estimate uncertainty ranges, which still likely underestimated realistic ranges because of neglected uncertainties in factors impacting calibrated $H_0$ such as the seasonality effect on exposure. The close-to-optimal $H_0$ were combined with a range in external input $\gamma '$ varied from 0 to 3 10$^{3}$ (thus two times the reference value, see Tab. S2) to calculate the corresponding uncertainty in model trajectories.
(B) Reference settings for non-zero disintegration of readiness ($r_H>$0) were applied in all subsequent experiments apart of a single run without decline in *H* ($r_H$=0 in Eq. (4), thus *H*=$H_0$).
(C) The calibration in (A) was repeated using the full data set; for the period from late December (2020) to mid January (2021), the RMS error was weighed ten times higher than that for the preceding period in order to achieve a reconstruction at elevated accuracy of the second wave. Also, three global settings of the reference run were systematically calibrated for each region: disintegration rate $r_H$, disintegration date $t_{\mathrm {reset}}$, and external input $\gamma '$.
(D) A series of 1.5-year simulations were run across the 20 regions as *H* was systematically increased from the regional reference value. Import rate $\gamma '$ and disintegration rate $r_H$ were set zero.
(E) Model sensitivities were assessed for two regions (Louisiana and Belgium) by varying 12 parameters 50% up and down from their reference value in Tab. S2.

## Supplementary Information

Supplementary Information.

## Data Availability

The code required to produce all model results is available at: https://github.com/kaiwirtz/CovidSocMod.
