# Supplementary material for: Changing readiness to mitigate SARS-CoV-2 steered long-term epidemic and social trajectories
Source: Sci Rep. 2021 Jul 6;11:13919. doi: 10.1038/s41598-021-93248-y (PMC8260599; doi:10.1038/s41598-021-93248-y)
Supplement: Supplementary file 1 — Supplementary Information. [file 41598_2021_93248_MOESM1_ESM.pdf]

Supporting Online Material

for

"Changing readiness to mitigate *SARS-CoV-2* steered long-term epidemic and social trajectories"

Kai Wirtz

- Supplementary Tables S1 to S2
- Supplementary Text
- Supplementary Figs. S1 to S11

Table S1: Regional characteristics: mean latitude (^{o}N), annual minimum and maximum temperature $T_{\rm min}$ and $T_{\rm max}$ (^{o}C), resp., median age A (yr) [1], relative proportion h' (%) of people above 65yr living in a mixed household [2], correction factor of mortality due to excess deaths f_M, initial thread awareness Δt (d), base human value H_0 (10^4), and relative immunity $\tilde{S}$=1 $-$ S (%) by May and Dec 2020. Temperature climatology (averaged Feb and Aug temperature 1994-2014) was available for US states [3] and non-US countries [4]. Regions were ordered with respect to mortality during the first wave.

country/state		lat.	$T_{\rm min}$	$T_{\rm max}$	A	h'	f_M	Δt	H_0	$\tilde{S}_{\rm May}$	$\tilde{S}_{\rm Dec}$
New Jersey	NJ	39.8	0.0	22.6	40.0	7	1.7	-15	1	13.9	71.0
New York	NY	43.0	-4.1	20.0	39.0	7	1.1	-13	5	9.1	64.4
Iran	IR	32.0	6.1	31.2	31.7	30	$<$15	-11	5	3.3	25.8
Belgium	BE	50.5	3.8	18.3	41.6	7	1.0	-15	7	5.8	49.8
Massachusetts	MA	42.4	-2.7	20.4	39.5	7	1.4	-12	2	6.6	64.9
Italy	IT	42.5	6.6	23.4	46.5	15	1.3	-19	10	4.4	19.6
United Kingdom	UK	54.0	4.4	15.1	40.6	7	1.2	-16	12	5.5	30.5
Switzerland	CH	47.0	-0.9	14.8	42.7	7	1.2	-9	6	2.2	28.7
Louisiana	LA	30.4	11.3	27.6	37.3	7	1.4	-7	2	7.2	59.1
Michigan	MI	44.2	-5.5	19.6	39.8	7	1.2	-12	3	4.3	53.6
Spain	ES	40.0	7.6	23.2	43.9	21	1.2	-11	16	5.3	27.8
Indiana	IN	40.3	-1.0	22.6	37.8	7	1.4	-9	4	3.0	56.7
France	FR	46.0	5.0	19.5	41.7	6	1.1	-11	6	3.1	42.3
Sweden	SE	62.0	-7.3	13.7	41.1	2	1.0	-13	4	4.1	29.8
Georgia	GA	33.2	9.4	26.3	36.8	7	1.4	-5	4	2.6	35.8
Netherlands	NL	52.3	3.8	17.8	42.8	3	1.2	-10	10	3.1	25.8
Portugal	PT	39.3	10.2	22.7	42.6	17	1.1	-9	14	0.9	16.4
Germany	DE	51.0	1.6	18.2	47.8	3	1.0	-11	13	0.5	8.9
Ireland	IE	53.0	5.7	14.9	37.8	13	1.2	-15	16	3.1	29.3
Washington	WA	47.8	2.8	18.3	37.7	7	1.4	-12	13	1.8	11.8

Table S2: Parameters, auxiliary variables, and dynamic state variables (SV) of the model.

	Description	Value	Unit
α_i	attack rates	0.3-1	
a_y	amplitude seasonal fluc.	0.2	
A	median population age		yr
$\widetilde{A}_i$	mean age of cohort i		yr
β_0'	initial β		d^{-1}
β_{ij}	age-spec. transmission rates	SV	d^{-1}
$\beta_{ij,0}$	base & initial values of β_{ij}		d^{-1}
C	social cost		d^{-1}
c_H	reduction factor of H	40	
δ	responsiveness	$1.7\ 10^3$	d^{-2}
Δt	initial thread awareness	Tab. S1	d
e	effective exposure		
ϵ	exposure sensitivity	1.6	
e_b	behavioral exposure		
e_E	environmental exposure		
f	mobility fluctuation		
γ_i	external input		d^{-1}
γ'	specific external input	$1.5\ 10^{-4}$	

	Description	Value	Unit
h_{ij}	household contr. of β_{ij}		
H	mitigation readiness		
H_0	base/initial value H	Tab. S1	
I_i	infected fraction	SV	
L	total loss		d^{-1}
M	COVID-19 mortality rate		d^{-1}
m_{ij}	contact rates		d^{-1}
φ_i	age distribution		
Q_{10}	IFR incr. per 10yr age	3	
r	recovery rate	0.08	d^{-1}
r_H	disintegration rate of H	0.013	d^{-1}
r_b	learning rate	0.05	d^{-1}
SD	social distance		d^{-1}
S_i	susceptibles	SV	
t_{reset}	first day of disintegration		d
θ	travel coefficient	0.35/1	
ω_i	infection fatality ratio (IFR)		
ω_7	IFR of oldest group	0.15	

S1 Age effects on infection fatality ratio and attack rate

Regional age distributions φ_i were interpolated using the median age A from Tab. S1 and the age distributions of the youngest population (Iran, φ_i^0, with median age A^0) and oldest population under consideration (Germany, φ_i^1, with median age A^1):

$$\varphi_i = (1-f)\,\varphi_i^0 + f\,\varphi_i^1 \qquad \text{with} \quad f = \frac{A - A^0}{A^1 - A^0} \tag{S1}$$

Age-specific infection fatality rates (IFR) ω_i increase in a non-linear way with the medium age $\widetilde{A}_i$ in each cohort, $\widetilde{A}_i \in \{10, 25, 40, 55, 65, 75, 90\}$. Compromising regional studies on the age dependency of case or infection fatality ratios [5, 6, 7, 8], I assume an increase by a factor Q_{10}=3 every ten years of lifetime:

$$\omega_i = \omega_7 \cdot Q_{10}^{(\widetilde{A}_i - \widetilde{A}_7)/10}$$

where the IFR of the oldest group ω_7 derives from reported estimates ranging from 0.1 to 0.16[5, 9, 10, 11]. Also attack rates α_i are reported to increase with age. Young individuals have a lower α_i than the elderly: they are not only less susceptible, but also less contagious [12]. Averaging over results from two studies [13, 14] yields the attack rates α_1=0.35, $\alpha_{2,3,4}$=0.5 and $\alpha_{5,6,7}$=1.

S2 Evaluation of marginal costs and benefits

Marginal costs and benefits of social distancing are in Eq.(7) represented by three pressures that in the model determine responses of societies coping with a deadly virus. The first pressure is generated by the numerous costs caused by social distancing, and mathematically reads as the partial derivative of social cost C with respect to transmission rates β_{ji}. Using Eqs.(2)–(4) we obtain a term describing relaxation to the pre-pandemic societal life ($m_{ij,0}$):

$$\frac{\partial C}{\partial \beta_{ji}} = 2\,H^{-1} \cdot \frac{\varphi_i\,\varphi_j}{\beta_{ij,0}} \cdot \Big(m_{ij,0} - m_{ij}(\beta_{ij})\Big)$$

The second pressure describes the risk of individuals to become infected and to die depending on their contacts. From Eq.(3) we have:

$$\frac{\partial M}{\partial \beta_{ij}} = \omega_j\,e\,I_i\,S_j \tag{S2}$$

This pressure increases with higher abundance of infected hosts in the interacting group (I_i), the size of the (susceptible) target age group (S_j), and with its IFR ω_j.

However, the two antagonistic pressures at the level of individual age groups cannot satisfactorily solve the regulation problem for younger age-groups because of vanishing ω_j and $\frac{\partial M}{\partial \beta_{ij}}$. Therefore, a third term is introduced in Eq.(7) to account for societal pressure as societies seek to lower infection levels of the entire population to better protect risk groups. This motivates the "community-oriented" derivative with respect to aggregated variables in Eq.(7), which contains two terms: First, the marginal dependence of total mortality on total infection density ($\mathrm{d}M/\mathrm{d}I$) equals the IFR. Contrary to the direct partial derivative Eq.(S2), the IFR has to be averaged over all groups: $\sum_k \omega_k\, \varphi_k$. Secondly, the marginal dependence of total infection density I on changes in β_{ji} ($\mathrm{d}I/\mathrm{d}\beta_{ji}$) is estimated assuming a steady state balance between infected cases (I_i) and transmission rates (β_{ji}) derived from from Eq.(1).

$$I_i = \frac{\beta_i\, S_i}{r} = \sum_j \frac{e\, \beta_{ji} I_j\, S_i}{r}$$

$$\frac{\mathrm{d}I}{\mathrm{d}\beta_{ji}} = \frac{e\, I_j\, S_i}{r}$$

$$\frac{\mathrm{d}M}{\mathrm{d}I} \cdot \frac{\mathrm{d}I}{\mathrm{d}\beta_{ij}} = \mathrm{e}^{\overline{\beta}\Delta t} \sum_k \omega_k\, \varphi_k \cdot \frac{e\, I_j\, S_i}{r} \tag{S3}$$

The ratio $\beta_i\, S_i / r\, I_i$ is known as reproductive number ("R"), which has often guided decision-making during the *SARS-CoV-2* pandemic. Eq.(S3) accounts for a lag period between infection and death, which influenced the estimated case fatalities during the exponential spread phase [15]. The mortality rate M in Eq.(3) expresses the actual death toll after a latency, symptomatic, and hospitalization period of approximately 21 days [15]. At any time, societies hence perceive a daily mortality which is by the factor $\mathrm{e}^{-\overline{\beta}\cdot 21d}$ smaller than the statistically projected one ($\overline{\beta}$ is the temporal average of recent mean transmission rates). The lag time difference also quantifies the capability to exponentially extrapolate from current case numbers to future mortality rates, which motivates the definition of the "thread awareness" Δt in Eq.(S3). Δt is in this study only used in the initial part of the simulation as calibration parameter to achieve a synchronous onset of lockdowns across regions (see "Numerical experiments" in Methods). A negative Δt corresponds to retarded and indecisive responses by decreasing the community pressure $\frac{\mathrm{d}M}{\mathrm{d}\beta_{ij}}$ responsible for risk reduction.

S3 Seasonal mobility cycle

Baseline contact rates ($m_{ij,0}$, Eq.(4), Eq.(S2)) are here assumed to vary with two seasonal factors. First, these changes can reflect the proportion of outdoor vs. indoor activities dependent on thermal comfort as quantified by the temperature function f_T:

$$f_T = \mathrm{e}^{-x^2/2} - \frac{1}{2} \qquad \text{with} \quad x = \frac{T - 20^o\mathrm{C}}{5^o\mathrm{C}} \tag{S4}$$

At neutral temperature around 20°C, maximized outdoor contacts will increase f_T (f_T=1/2) while at very low or high temperature ($T \ll 10^o$C or $T \gg 30^o$C, respectively), this index will be negative (f_T=-1/2). Air temperature T for simplicity follows a trigonometric function including a time lag since in the Northern Hemisphere T is usually lowest around early February:

$$T = T_{\min} + (T_{\max} - T_{\min}) \cdot \sin^2\left(\frac{(t - 35\mathrm{d})\,\pi}{365\mathrm{d}}\right) \tag{S5}$$

with simulation time t counted from January, 1st. Minimal and maximal annual temperatures $T_{\min}$ and $T_{\max}$, respectively, are given in Tab. S1.

As complementary seasonal driver the model includes the annual light cycle quantified by relative daylength DL. DL is calculated using an astronomical formula with time t and the mean latitude of a region (Tab. S1) as inputs. Similar to f_T, the seasonal light index f_{DL} describes how longer days permit more activities and contacts:

$$f_{\mathrm{DL}} = \mathrm{atan}\Big(2 \cdot (\mathrm{DL} - 0.5)\Big) \tag{S6}$$

where the *atan* function smoothes a step function: for locations at higher latitude such as Sweden f_{DL} approaches -1/2 when DL is small during winter and 1/2 at large DL during summer (most people do not stay outside about 20h per day at summer solstice).

The temperature based index f_T and the light index f_{DL} are simply added up to yield the overall seasonal mobility fluctuation f:

$$f = 1 + a_y \cdot (f_T + \theta f_{\mathrm{DL}}) \tag{S7}$$

The amplitude a_y is estimated from reported seasonal differences in outdoor activities [16] and from aggregated mobile phone data for 2016 [17]. However, the latter data-set reveals much greater seasonality in Europe compared to the US (SI-Figs.3 and 5 in [17]). A much lower seasonal

amplitude and maximum intensity in USA compared to European states has also been reported for traveling [18]. Therefore, the coefficient θ in Eq.(S7) is set to one for European states but to a lower value elsewhere (see Tab. S2).

S4 Contact vs. transmission rates and reciprocity condition

When considering age-specific mixing patterns, one has to distinguish between transmission rates (β_{ij}) and contact rates (m_{ij}). As introduced in the main text, β_{ij} describe the encounter probability per individual, whereas m_{ij} the one per age group. The reciprocity condition ($m_{ij} = m_{ji}$) with Eq.(2) transforms to

$$\varphi_i \beta_{ij} = \varphi_j \beta_{ji}$$

Hence, from the 7×7 matrix coefficients β_{ij}, only 28 are independent and used as model variables. To ensure reciprocity at any time, all terms in the evolution equation Eq.(7) are calculated with swapped indices, thus for both β_{ij} and β_{ji}(=$\beta_{ij}\varphi_i/\varphi_j$), and then averaged.

S5 Initial and base transmission rates

Business-as-usual (BAU) transmission rates $\beta_{ij,0}$ were retrieved starting from reported (time independent) contact matrices $\overline{m}_{ij,0}$, which were available for European countries from the POLYMOD project [19]. In that study, relative proportions of different interaction fields were: 0.3 for households, 0.18 for schools, 0.19 for workplaces and 0.33 for the general community. Analysis of accompanying studies [19, 20] suggests that this approach underestimated interaction with friends. I therefore added a 30% and 12% contribution in intra-group contacts for below 30yr and above 30yr, respectively [21]. The resulting contact matrices emphasize the diagonal elements (Fig. S11). Contact matrices of non-European countries (Iran, USA) were set equal to the one of UK and transformed to base contact rates $\overline{m}_{ij,0}$ using the interpolated age distribution (φ_i) described in Sec. S1. Time habits in BAU mixing were considered by multiplication with the seasonal forcing function f given in Eq.(S7):

$$m_{ij,0} = f\,\overline{m}_{ij,0}$$

The date when the reported daily COVID-19 associated death toll meets the threshold M=7·10^{-7}d^{-1} defines the simulation start for each region (see Methods). Then, the initial transmission $\beta_{ij,0}$ (Eq.(2))

$$\beta_{ij,0} = \beta_0' \, \frac{\alpha_i \alpha_j \, f \, \overline{m}_{ij,0}}{\varphi_i}$$

is tuned such that the temporal increase in mortality (proportional to the $\beta_{ij,0}$) during ten days after this onset corresponds to the reported mortality increase. Thus, the proportionality factor β_0' is specific for each region.

S6 Minimal contacts depending on household structure

BAU transmission rates $\beta_{ij,0}$ not only define upper target values for the adaptive dynamics in β_{ij} in Eq.(S2), they also constitute reference values for lower bounds of social distancing. These lower bounds are especially relevant in simulations at high H since real subsistence activities and household contacts cannot be shut-down entirely:

$$\beta_{ij} \geq \max\{h_{ij}, h_{\min}/h'\} \cdot \beta_{ij,0}$$

Relative household contributions h_{ij} to base transmission rates $\beta_{ij,0}$ not only reflect the global relative weighing factor (0.3, see above) but also the variable age-specific intensity of household interactions. The coefficients h_{ij} were reconstructed from [22], with age-dependent co-habitation values of h_{11}=0.35, h_{ii}=0.45 for i=2. . .5, and h_{66}=h_{77}=0.65, whereas a higher value of h_{13}=0.75 is estimated for the intergenerational contact between children and their parents. Apart of children-parents constellations, intergenerational households also comprise the elderly. The relative proportion h' of elderly above 65yr that live in a mixed household has been reported to vary between European countries from 1.6% (SE) to 20.6% (ES) [2] (Tab. S1). h' contributes to few non-diagonal coefficients h_{ij} of the household contact distribution: h_{16}=h_{36}=h_{37}=h_{47}=h'. With a higher fraction of elderly in mixed household I assume a lower relevance of caring infrastructure and also more small-scale solutions for daily supply. Therefore, the subsistence related proportion of contact rates in Eq.(S6) is a constant ($h_{\min}$=1/2) divided by h'. For example, food supply, nurseries, and health care facilities can hardly be (en)closed when no care services are provided by families.

In the calculation of the mobility index,

$$\text{Mobility} = \sum_{ij} \Big(m_{ij} - h_{ij} m_{ij,0}/2 \Big) \cdot \varphi_i \varphi_j \tag{S8}$$

for simplicity half of household contacts $h_{ij} m_{ij,0}$ are subtracted from contact rates m_{ij} since many of these contacts will not require routing requests by mobile phone users. Analogous to the mobility index from mobile phone data provided by Google and Apple, the weighed sum was normalized by the weekly average directly after simulation start.

S7 Spurious and imported cases

Within each age class, infection density I_i is set to zero when I_i falls below the threshold of 10^{-7}, which corresponds to one individual in a population of 10 million (to avoid unrealistic residual infection levels). This condition was only met in few scenario runs with very low SCV.

At low infection density, the (small) external input rate γ_i in Eq.(1) gains quantitative importance [23]. The term describes local re-intrusion of cases by business or touristic travelers:

$$\gamma_i = \gamma' \, \varphi_i \, \overline{\beta}_i \tag{S9}$$

with the travel coefficient $theta$ (Sec. S3), a specific global input rate γ', the relative size of the age group φ_i, and contact propensity quantified by the averaged transmission rate $\overline{\beta}_i$=$\sum_j \beta_{ji}/7$. Lacking regional differences, γ_i is set to zero before the first lockdown to avoid artifacts in the initial model dynamics.

S8 Environmental exposure

The variable exposure e in Eq.(1) describes the effective reduction in transmission independently from social distancing and is in this approach split-up into the two factors behavioral exposure (e_b, see Eq.(8)) and environmental conditions (e_E)

$$e = e_b \cdot e_E$$

Environmental exposure e_E also carries behavioral aspects but triggered by ambient conditions. Due to the pre-dominant aerosol transmission of *SARS-CoV-2*, outdoor contacts are considered

much less contagious so that the effect of temperature driven increase in activity (see above) can be reverted if contacts are shifted from indoor to outdoor micro-environments. These opposing effects may also explain lacking clear temperature correlations. However, many studies point to an at least moderate decrease in transmission efficiency with rising temperature [24, 25, 26, 27, 28]. Underlying these findings may be decreased viability of *SARS-CoV-2* on surfaces at higher temperature [29, 30]. More pronounced are effects of UV light on viral activity [31, 32]. In short, summer conditions ($f > 1$, see Sec. S3) have a strong negative impact on transmission efficiency, both indirectly and directly. As a consequence of direct ambient forcings, seasonal fluctuations not only revert their sign compared to the formulation f in Eq.(S7), but also increase in magnitude:

$$e_E = 1 - 2a_y \cdot (f_T + \theta f_{\mathrm{DL}})$$

The increase of activities and contacts in warmer and lighter seasons is thus more than compensated by a higher reduction in environmental exposure e_E. This setting and parametrization was already condensed in a preceding model study to a net effect of a maximal temperature variation on transmission of around 25% [33].

S9 Model limitations and uncertainties

Uncertainties of this reconstruction first pertain integrated data. The mortality data only roughly account for excess deaths or for region-specific or shifting attribution criteria such as for Ireland on Apr, 24. Although trends in the mobility data from Apple could be confirmed by similar products provided by Google (not shown) or national providers [34], the association between routing requests and real physical contacts remains approximate. Yet, aggregate mobility seems to be a better proxy than implementation dates of certain policies since their containment effect can differ between countries [35]. Future model versions should include an updated parametrization and more details from existing SIR models such as spatial structure [36, 37, 38], re-infection [39], variable viral persistence and recover [40], or age dependency of infectiousness [41]. The aggregate nature of the societal model should be dis-entangled to better resolve partially independent dynamics in political, economical, cultural, and psychological constituents of behavioral exposure and mitigation readiness. In particular, the rather heuristic logistic equation for describing

disintegration of cohesion and accumulation of fatigue should be replaced by more mechanistic approaches. Aggregation also infers a lower spatial limit of model applications as in local communities more specific drivers can be expected to drive mitigation dynamics. The aggregation finally imposes a lower temporal scale as it neglects, among others regional differences in cultural conditioning. For example, in Ireland or Georgia, mitigation readiness likely dropped during Christmas, leading to massive mortality peaks, which were predicted by the model in terms of magnitude but with a relatively large time delay.

A sensitivity analysis demonstrated qualitative robustness of major outcomes (Fig. S10), but also revealed few major uncertainties such as for IFR or attack rates of the younger age classes, or for the few societal coefficients of the model. Certain model settings and assumptions will probably remain untested since changing behavior during crises situation is difficult to monitor. Due to the lower median age of the US compared to European countries (Tab. S1), the here presented selection of regions infers a weak correlation between median age and H, which may not be the case for emerging countries (e.g., see intermediate H of Iran).

Supporting figures S1–S11

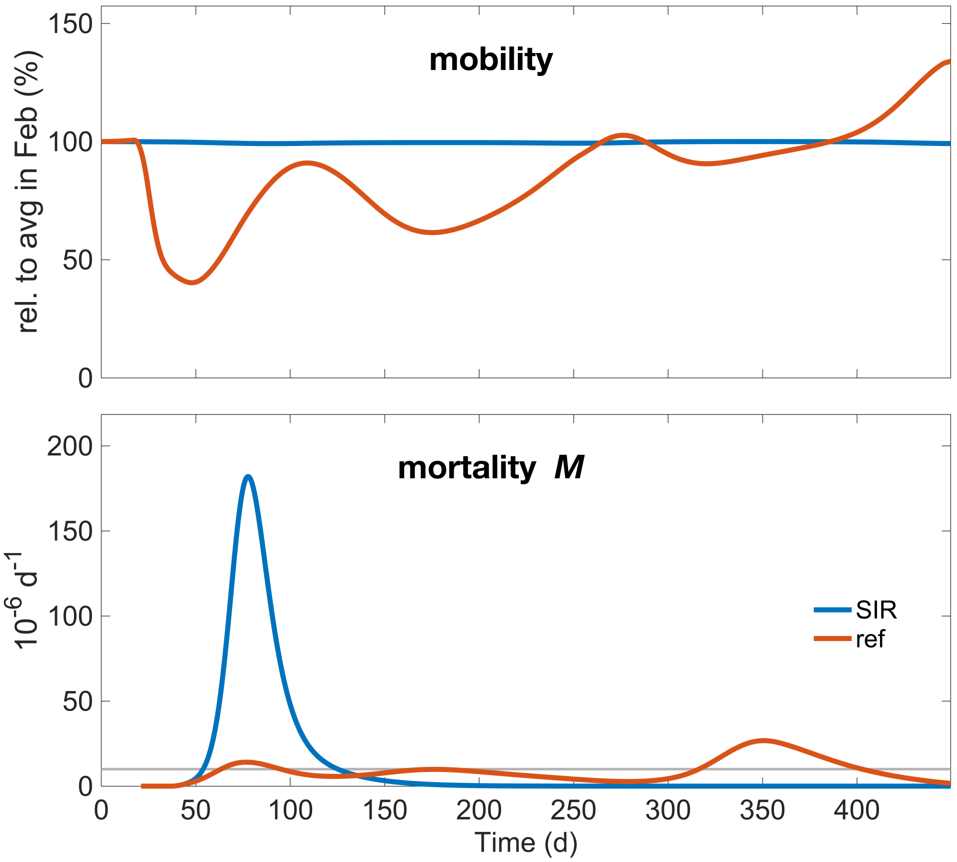

Figure S1: Mobility (top) and mortality rate (bottom) in the reference simulation for Germany (red line) compared with a conventional SIR simulation insofar lacking humany agency (specific contact and behavior adoption r_b=δ=0).

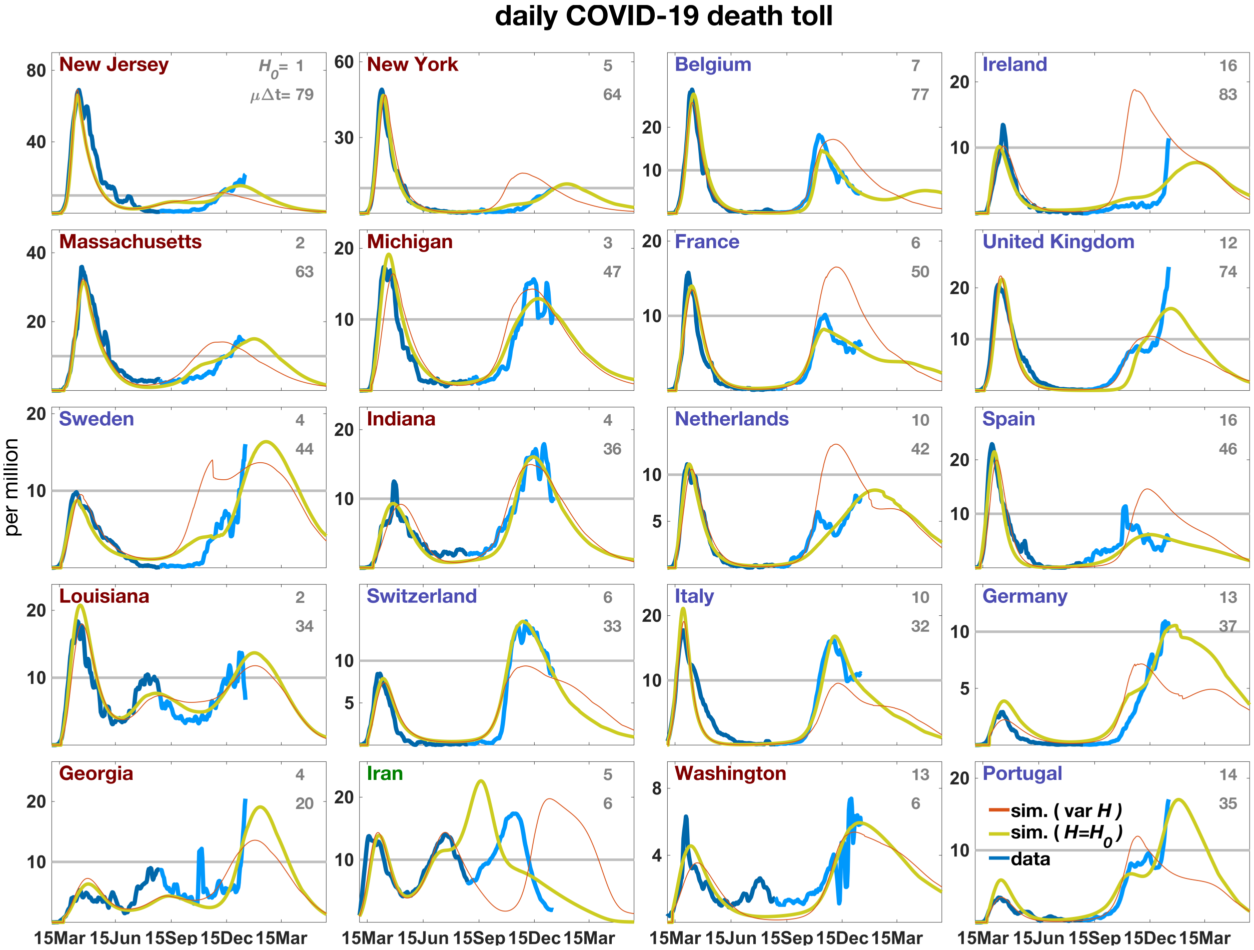

Figure S2: Mortality rate in the reference simulation (thin red line, Fig. 2) compared with reported data (blue line) and a simulation with extended calibration (olive line) where three parameters were fitted for each region separately using the full data set instead of only the first 180d. Calibrated values for disintegration rate r_H (10^{-2} d^{-1}, Eq.(5)), external input γ' (10^{-4}, Eq.(1) and S9), and disintegration date $t_{\rm reset}$ (day in 2020, Eq.(5)) are: BE:1.1/0/321, FR:0.5/2/193, DE:4.4/2/289, IR:3.3/0/193, IE:3.3/12/289, IT:4.4/2/225, NL:2.2/5/257, PT:3.3/2/289, ES:1.7/12/161, SE:3.9/5/289, CH:3.3/2/225, UK:3.9/0/289, GA:2.8/0/289, IN:2.8/12/225, LA:2.2/5/257, MA:2.8/0/289, MI:1.7/10/225, NJ:5.0/0/289, NY:2.8/12/289, WA:4.4/12/257.

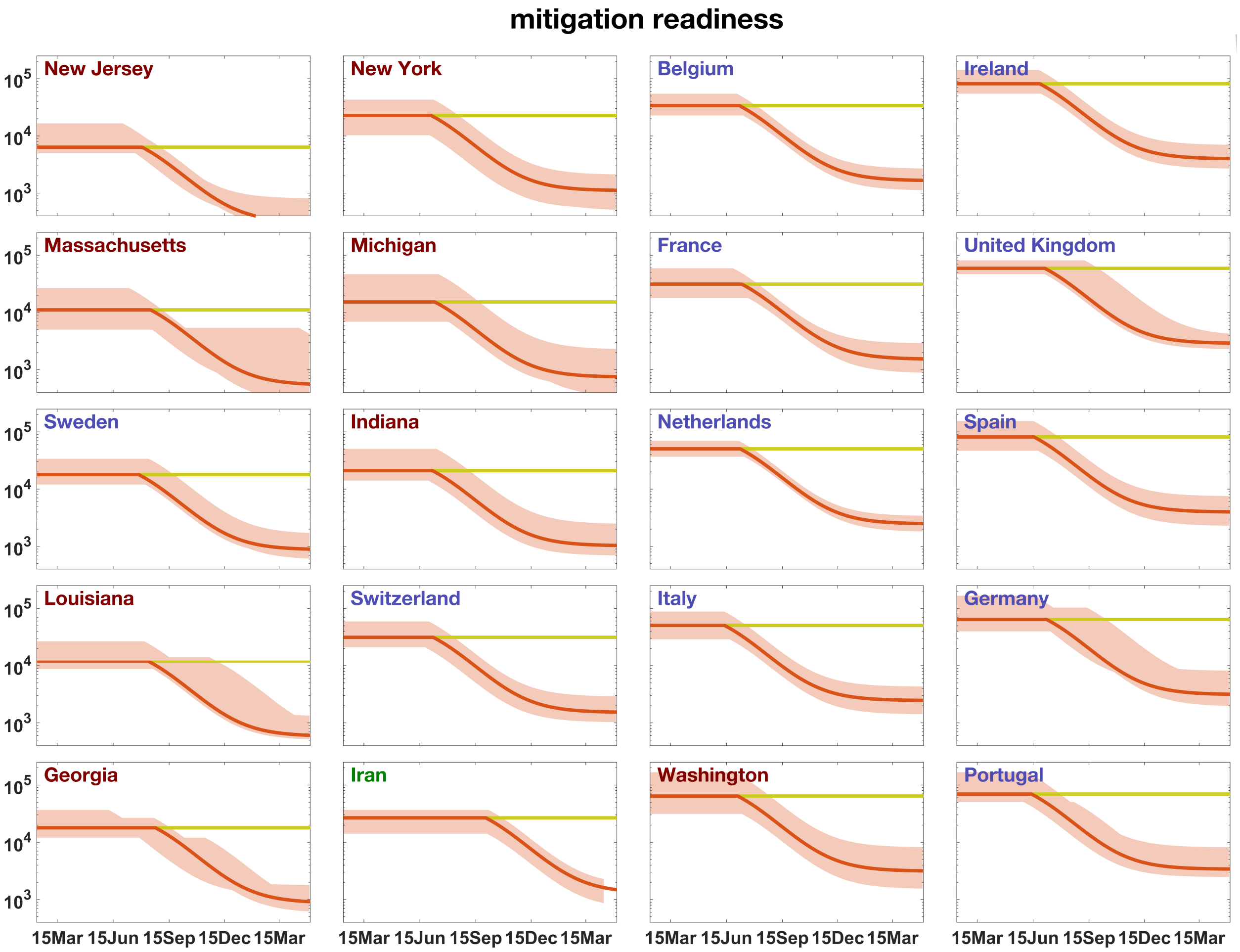

Figure S3: Mitigation readiness H, here defined as the value of human lives versus BAU contact rates, in the two model variants: either with disintegration of H from its regional base value H_0 ($r_H > 0$ in Eq.(5), red line), or without disintegration (olive line). Uncertainty ranges in the variant with disintegration (shaded area) derive from a released selection criteria during parameter calibration (see Methods).

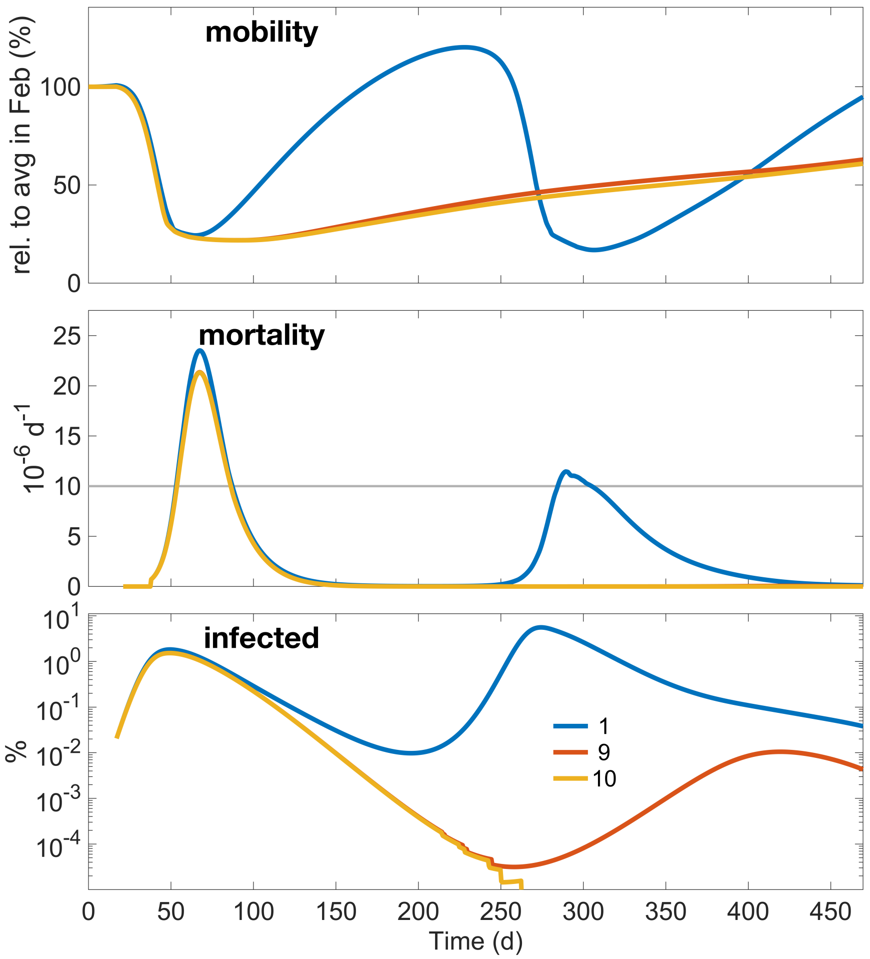

Figure S4: Comparison of aggregated variables in simulations for Belgium where the mitigation readiness is kept constant within the run (H=H_0), but varied for each run from the reference value (H_0=6.8 10^4). Three proportionality factors were used: 1 (blue line), 9 (red), and 10 (orange). From top: mobility from Eq.(S8), mortality M, and total fraction of infected individuals $I = \sum_i I_i$.

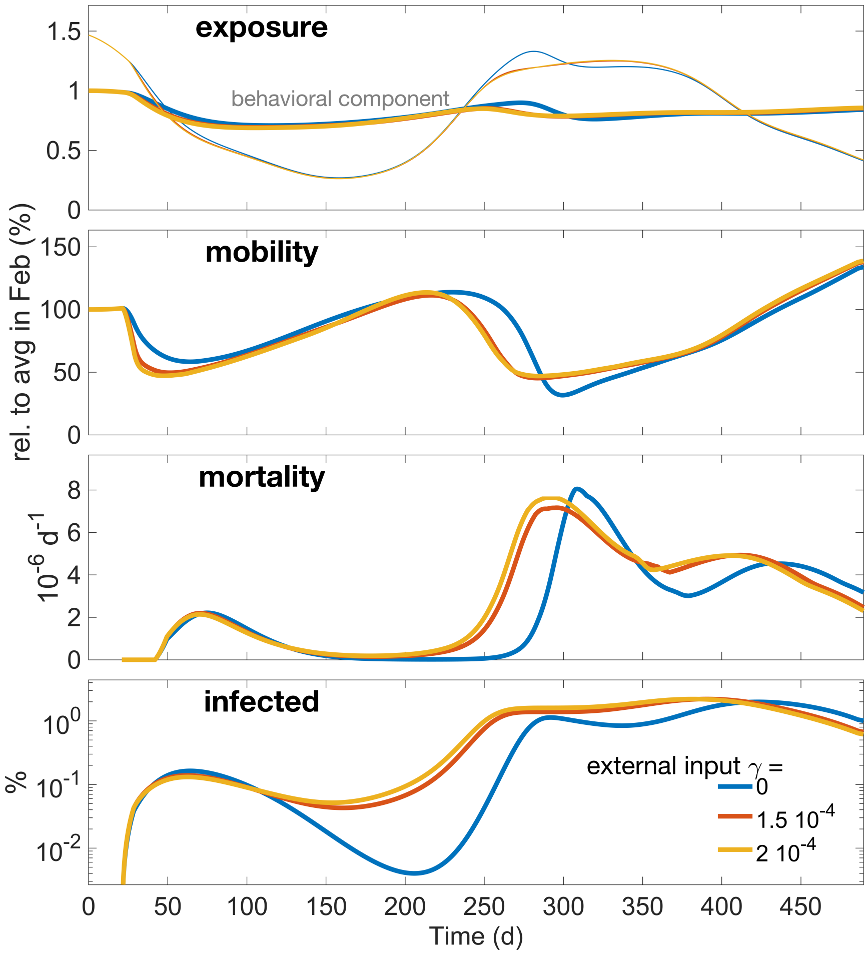

Figure S5: Aggregated variables in simulations where the reference value of the specific external input γ'=1.5 10^{-4} (red line) is varied either downward (γ'=0, blue line) or upwards γ'=2 10^{-4} (orange line). From top: effective exposure e (thin lines) and the behavioral contribution e_b (thick lines), mobility, mortality, and relative number of cases in the population.

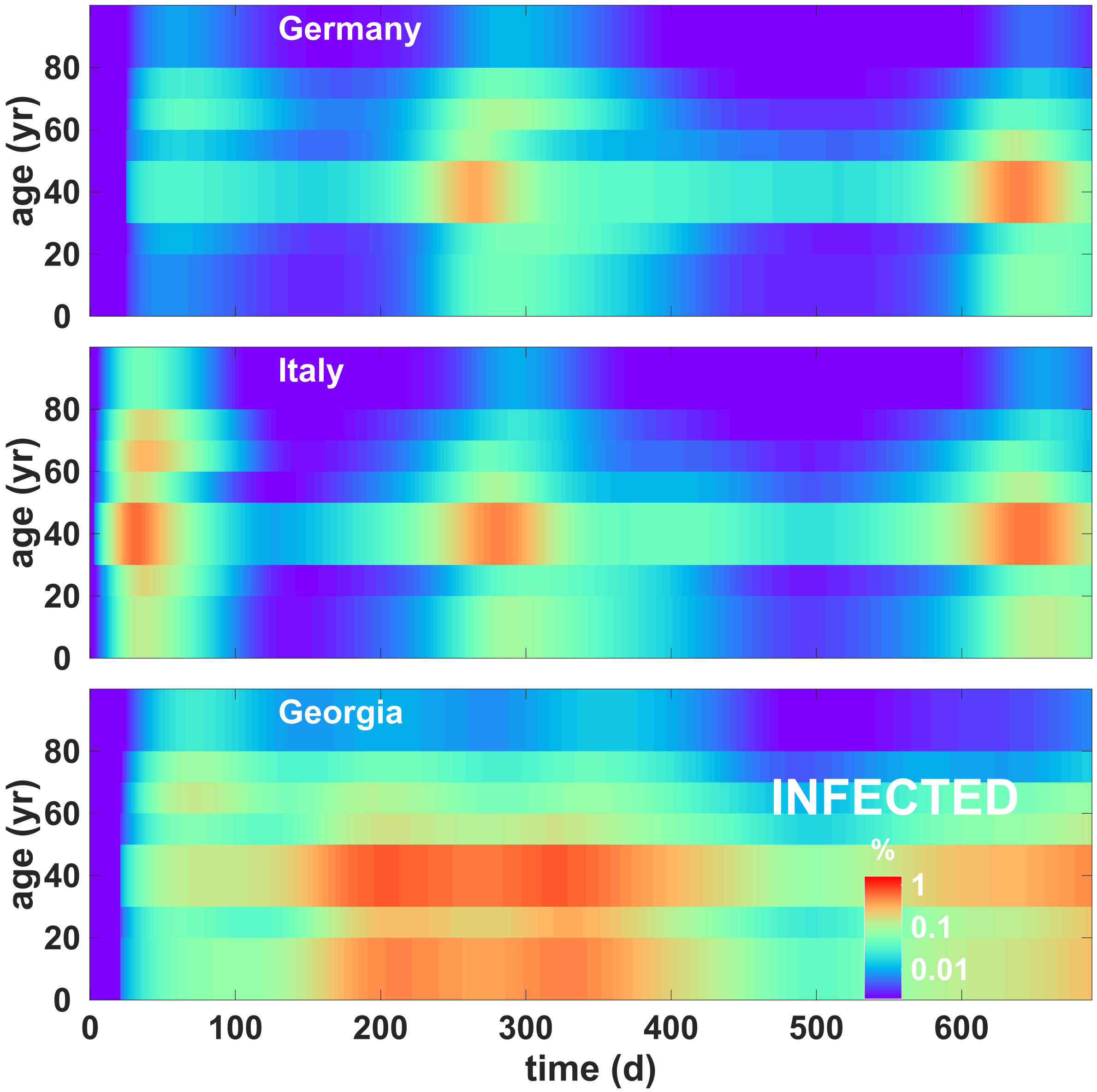

Figure S6: Time evolution of the relative density of infected people (I_i) over two years for all age classes in Italy, Germany, and Georgia.

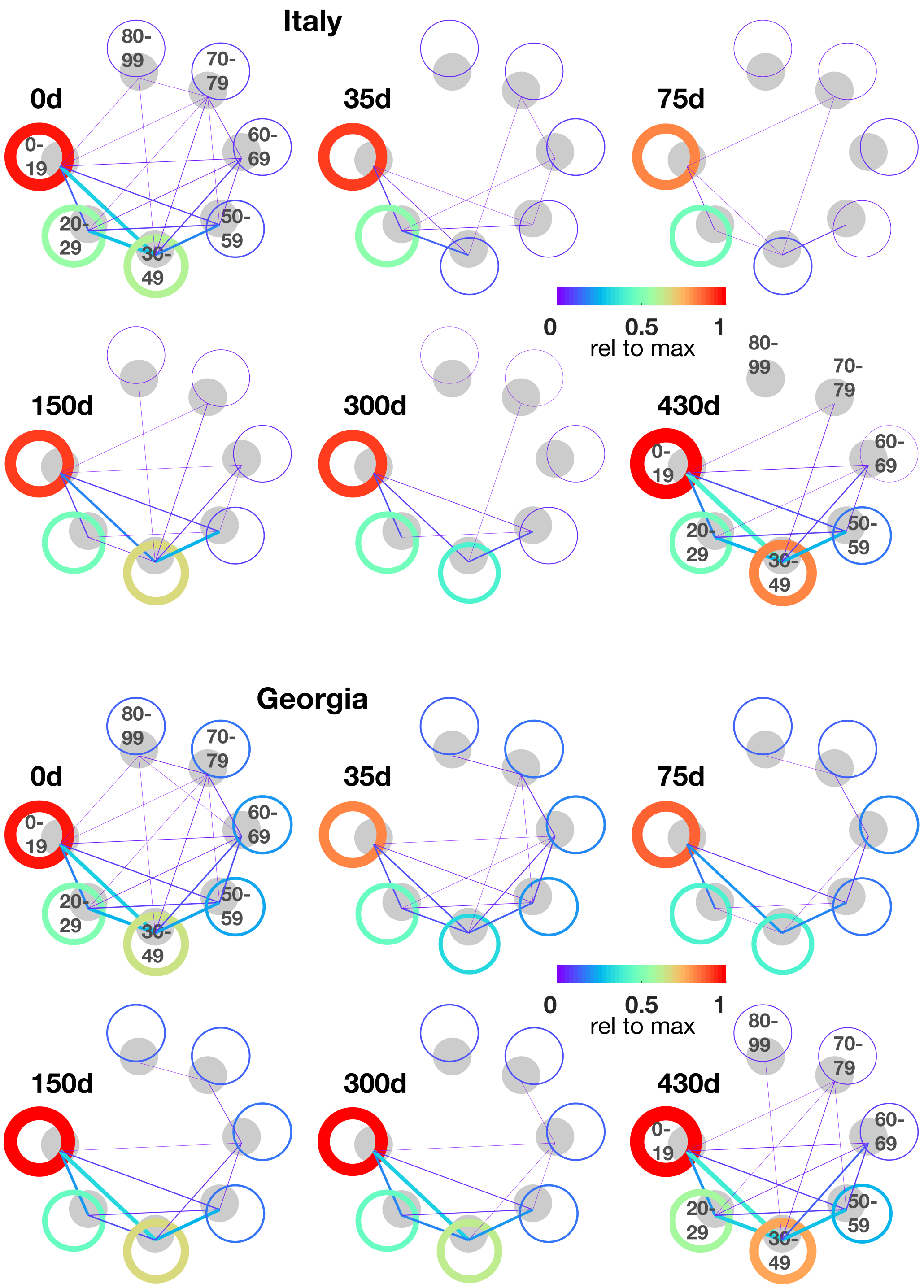

Figure S7: Age-specific interaction patterns ($\beta_{ij} \cdot \varphi_i$) at distinct times in Italy and Georgia. Contact intensity is normalized to the pre-pandemic interaction in the youngest age group and represented as color and line thickness.

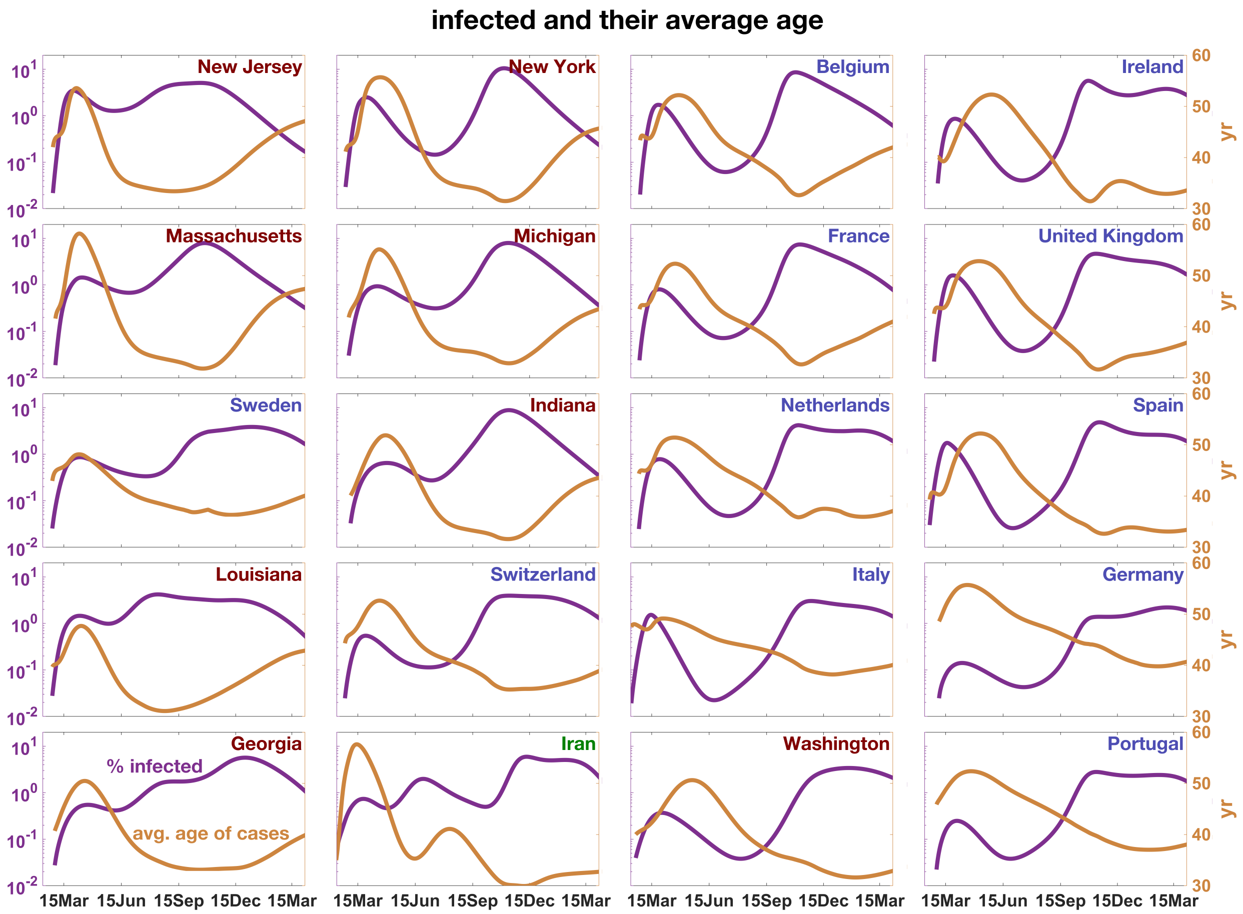

Figure S8: Simulated fraction of infected cases $I = \sum_i I_i$ (purple line) and their mean age (brown line) for the 20 study regions from Feb 2020 to Apr 2021 (reference run with $r_H > 0$).

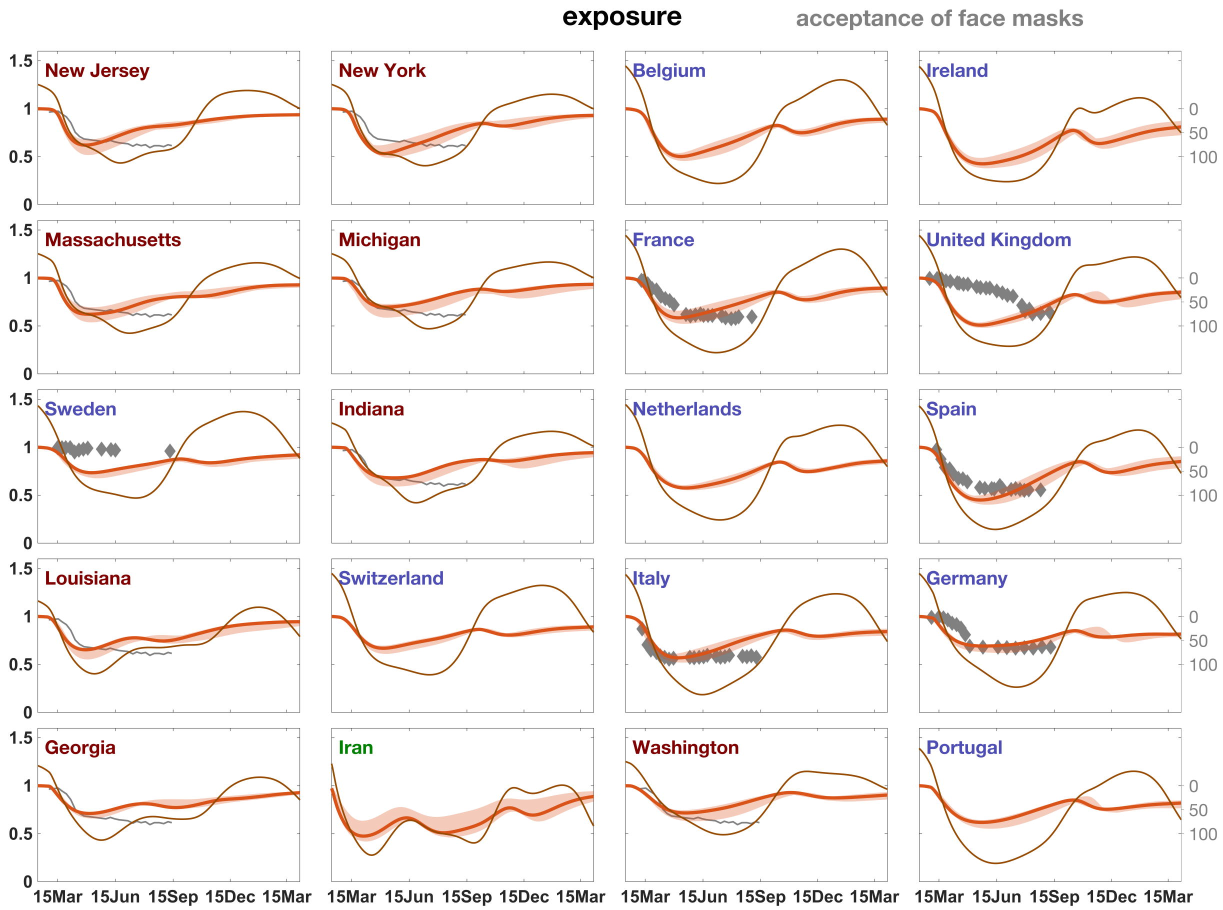

Figure S9: Behavioral exposure e_b (red line) and effective exposure, $e = e_b \cdot e_E$ (brown line), thus encompassing the seasonality of the environmental exposure e_E, simulated for all study regions. Behavioral exposure is tentatively compared with the survey data on the acceptance to wear face masks in the public [42] (grey dots; willingness in %). For the US, no data were available at state level. European countries are labelled in blue, US states in red.

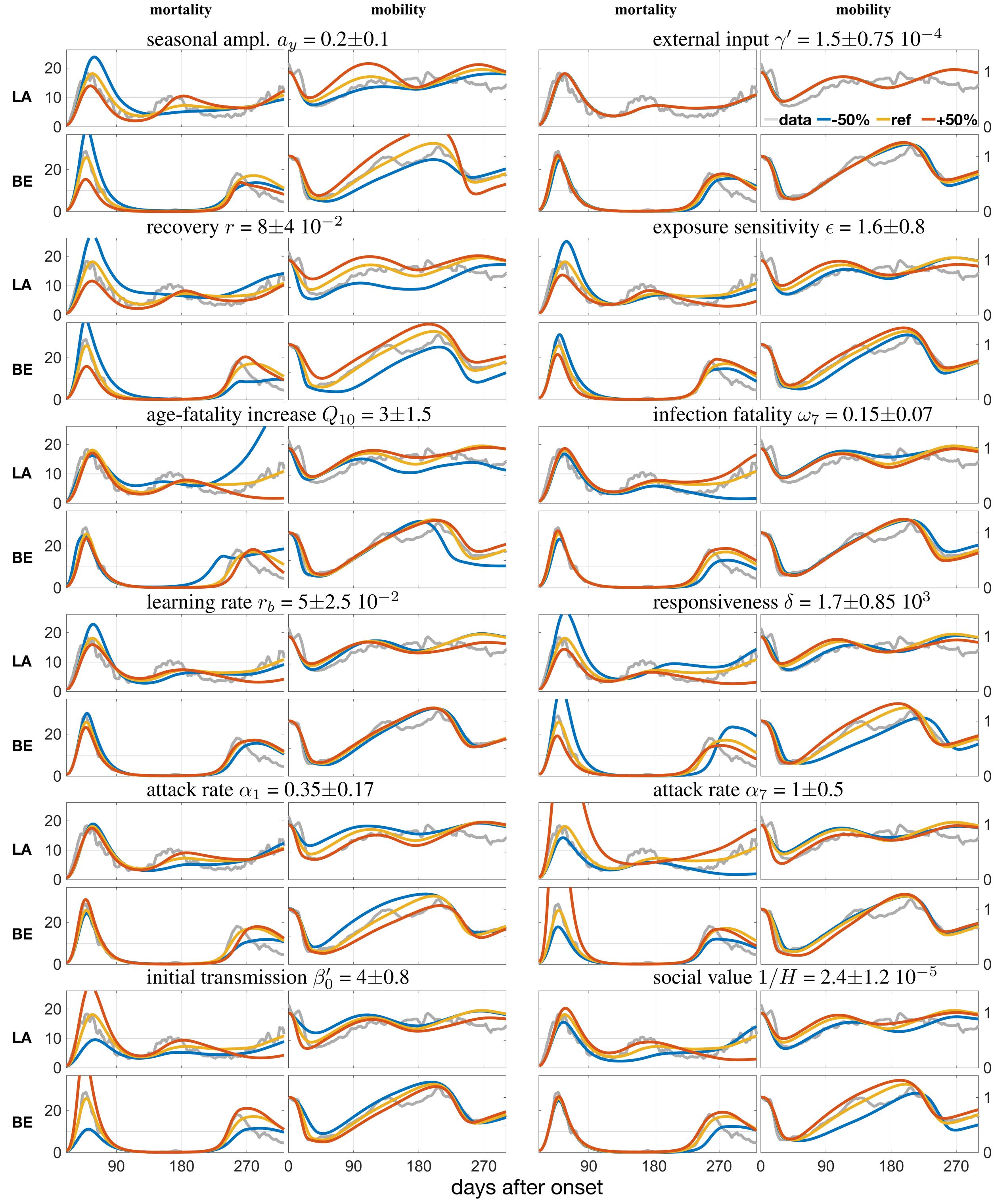

Figure S10: Sensitivity analysis: effect of individual parameter variations on two major model variables (mobility and mortality) for Belgium (BE) and Louisiana (LA).

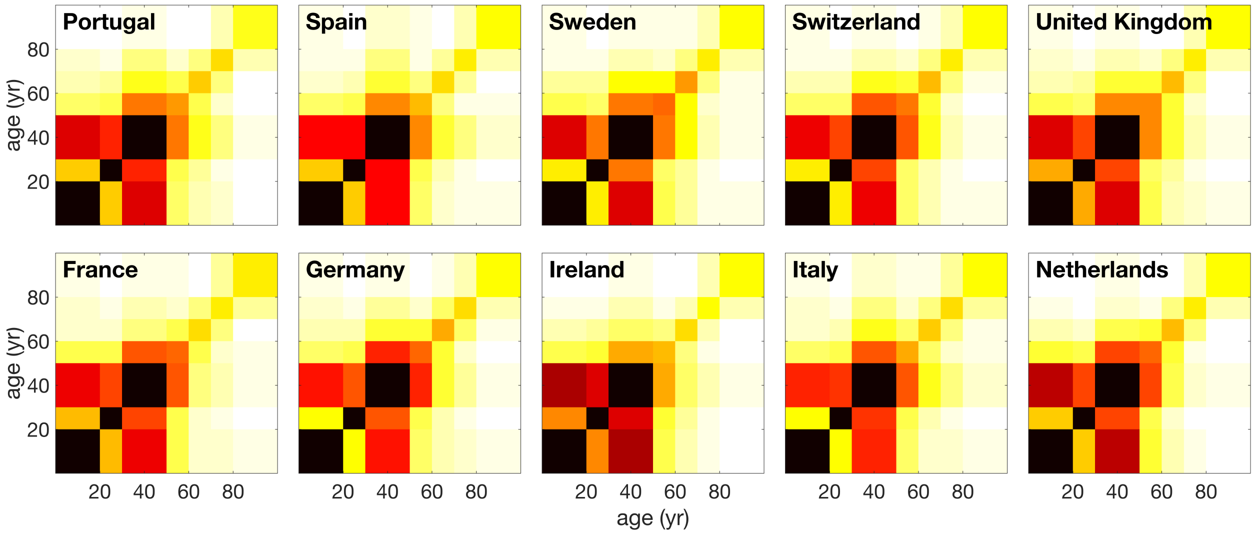

Figure S11: Initial and BAU contact matrices m_{ij} for ten European countries from POLYMOD data [19] after adding intra-group contacts with friends. Dark fields indicate strong mixing.

References

[1] CIA (2020) CIA world factbook, Technical report. https://www.cia.gov/library/publications/resources/the-world-factbook/fields/343rank.html.

[2] Iacovou M, Skew A (2010) Household structure in the EU, (ISER working paper 2010-10), Technical report.

[3] Weatherbase (2020), Technical report. https://www.weatherbase.com.

[4] NERC (2020) The NERC CEDA Archive, Technical report. http://crudata.uea.ac.uk/cru/data/hrg/cru_ts_3.23.

[5] Salje H, et al. (2020) Estimating the burden of SARS-CoV-2 in France. *Science*. doi:10.1126/science.abc3517.

[6] Onder G, Rezza G, Brusaferro S (2020) Case-fatality rate and characteristics of patients dying in relation to COVID-19 in Italy. *Jama* 323(18):1775–1776.

[7] European Centre for Disease Prevention and Control (2020) Rapid risk assessment: Coronavirus disease 2019 (COVID-19) in the EU/EEA and the UK–ninth update. https://www.ecdc.europa.eu/sites/default/files/documents/covid-19-rapid-risk-assessment-coronavirus-disease-2019-ninth-update-23-april-2020.pdf.

[8] Laxminarayan R, et al. (2020) Epidemiology and transmission dynamics of COVID-19 in two Indian states. *Science* 370(6517):691–697.

[9] Ward H, et al. (2020) Antibody prevalence for SARS-CoV-2 in england following first peak of the pandemic: React2 study in 100,000 adults. *medRxiv*. doi:10.1101/2020.08.12.20173690.

[10] Ferguson NM, et al. (2020) Imperial College COVID-19 Response Team. Impact of non-pharmaceutical interventions (NPIs) to reduce COVID-19 mortality and healthcare demand. *Imperial College London (Report)*. doi:0.25561/77482.

[11] Pastor-Barriuso R, et al. (2020) SARS-CoV-2 infection fatality risk in a nationwide seroepidemiological study. *medRxiv*. doi:110.1101/2020.08.06.20169722.

[12] Ludvigsson JF (2020) Children are unlikely to be the main drivers of the COVID-19 pandemic–a systematic review. *Acta Paediat.* doi:10.1111/apa.15371.

[13] Bi Q, et al. (2020) Epidemiology and transmission of COVID-19 in 391 cases and 1286 of their close contacts in Shenzhen, China: a retrospective cohort study. *Lancet*. doi:0.1016/S1473-3099(20)30287-5.

[14] Zhang J, et al. (2020) Age profile of susceptibility, mixing, and social distancing shape the dynamics of the novel coronavirus disease 2019 outbreak in China. *medRxiv*. doi:10.1101/2020.03.19.20039107.

[15] Baud D, et al. (2020) Real estimates of mortality following COVID-19 infection. *Lancet*. doi:10.1016/S1473-3099(20)30195-X.

[16] Leech JA, Nelson WC, T BURNETT R, Aaron S, Raizenne ME (2002) It's about time: a comparison of Canadian and American time–activity patterns. *J. Exposure Sci & Env. Epidemiol.* 12(6):427–432.

[17] Kraemer MU, et al. (2020) Mapping global variation in human mobility. *Nature Hum. Beh.* 4:800–810.

[18] Hawelka B, et al. (2014) Geo-located twitter as proxy for global mobility patterns. *Cart. Geogr. Inf. Sci.* 41(3):260–271.

[19] Fumanelli L, Ajelli M, Manfredi P, Vespignani A, Merler S (2012) Inferring the structure of social contacts from demographic data in the analysis of infectious diseases spread. *PLoS Comput.Biol.* doi:10.1371/journal.pcbi.1002673.

[20] Arregui S, Aleta A, Sanz J, Moreno Y (2018) Projecting social contact matrices to different demographic structures. *PLoS Comput. Biol.* 14(12):e1006638.

[21] Cornwell B (2011) Age trends in daily social contact patterns. *Res. Aging* 33(5):598–631.

[22] Prem K, Cook AR, Jit M (2017) Projecting social contact matrices in 152 countries using contact surveys and demographic data. *PLoS Comput. Biol.* 13(9):e1005697.

[23] Russell TW, et al. (2020) Effect of internationally imported cases on internal spread of COVID-19: a mathematical modelling study. *Lancet Pub. Health* 6(1):e12–e20.

[24] Lin J, et al. (2020) Containing the spread of coronavirus disease 2019 (COVID-19): Meteorological factors and control strategies. *Sci. Tot. Environ.* 744:140935.

[25] Ujiie M, Tsuzuki S, Ohmagari N (2020) Effect of temperature on the infectivity of COVID-19. *Int. J. Infect. Diseases* 95:301–303.

[26] Prata DN, Rodrigues W, Bermejo PH (2020) Temperature significantly changes COVID-19 transmission in (sub) tropical cities of Brazil. *Sci. Tot. Environment* p. 138862. doi:10.1016/j.scitotenv.2020.138862.

[27] Araujo MB, Naimi B (2020) Spread of SARS-CoV-2 coronavirus likely to be constrained by climate. *medRxiv*. doi:10.1101/2020.03.12.20034728.

[28] Wang J, Tang K, Feng K, Lv W, Wan F (2020) High temperature and high humidity reduce the transmission of COVID-19. *archIX*. doi:10.2139/ssrn.3551767.

[29] Riddell S, Goldie S, Hill A, Eagles D, Drew TW (2020) The effect of temperature on persistence of SARS-CoV-2 on common surfaces. *Virology Journal* 17(1):1–7.

[30] Biryukov J, et al. (2020) Increasing temperature and relative humidity accelerates inactivation of SARS-CoV-2 on surfaces. *MSphere* 5(4):1–9.

[31] Schuit M, et al. (2020) Airborne SARS-CoV-2 is rapidly inactivated by simulated sunlight. *J. Infect. Diseases* 222:564–571.

[32] Sloan A, et al. (2020) Simulated sunlight decreases the viability of SARS-CoV-2. doi:10.21203/rs.3.rs-37057/v1.

[33] Kissler SM, Tedijanto C, Goldstein E, Grad YH, Lipsitch M (2020) Projecting the transmission dynamics of SARS-CoV-2 through the postpandemic period. *Science* 368(6493):860–868.

[34] Glogowsky U, Hansen E, Schächtele S (2020) How effective are social distancing policies? Evidence on the fight against COVID-19 from Germany. *SSRN working paper*. doi:10.2139/ssrn.3619845.

[35] Hsiang S, et al. (2020) The effect of large-scale anti-contagion policies on the COVID-19 pandemic. *Nature*. doi:10.1038/s41586-020-2404-8.

[36] Pei S, Kandula S, Shaman J (2020) Differential effects of intervention timing on COVID-19 spread in the United States. *medRxiv*. doi:10.1101/2020.05.15.20103655.

[37] Yamana T, Pei S, Kandula S, Shaman J (2020) Projection of COVID-19 cases and deaths in the US as individual states re-open May 4, 2020. *medRxiv*. doi:10.1101/2020.05.04.2009067.

[38] Tian H, et al. (2020) An investigation of transmission control measures during the first 50 days of the COVID-19 epidemic in China. *Science* 368(6491):638–642.

[39] Malkov E (2020) Simulation of coronavirus disease 2019 (COVID-19) scenarios with possibility of reinfection. *Chaos, Solitons & Fractals* 139:110296.

[40] Wang S, et al. (2020) Modeling the viral dynamics of SARS-CoV-2 infection. *Math. Biosci.* 328:108438.

[41] Cevik M, et al. (2020) SARS-CoV-2, sars-cov, and mers-cov viral load dynamics, duration of viral shedding, and infectiousness: a systematic review and meta-analysis. *Lancet Microbe*. doi:10.1016/S2666-5247(20)30172-5.

[42] YouGov (2020), Technical report. https://in.yougov.com/en-hi/news/2020/04/20/yougovs-international-covid-19-tracker-reveals-cha.
